# Parkinson’s disease-associated alterations of the gut microbiome predict disease-relevant changes in metabolic functions

**DOI:** 10.1186/s12915-020-00775-7

**Published:** 2020-06-09

**Authors:** Federico Baldini, Johannes Hertel, Estelle Sandt, Cyrille C. Thinnes, Lorieza Neuberger-Castillo, Lukas Pavelka, Fay Betsou, Rejko Krüger, Ines Thiele, on behalf of the NCER-PD Consortium, Gloria Aguayo, Dominic Allen, Wim Ammerlann, Maike Aurich, Rudi Balling, Peter Banda, Katy Beaumont, Regina Becker, Daniela Berg, Sylvia Binck, Alexandre Bisdorff, Dheeraj Bobbili, Kathrin Brockmann, Jessica Calmes, Lorieza Castillo, Nico Diederich, Rene Dondelinger, Daniela Esteves, Jean-Yves Ferrand, Ronan Fleming, Manon Gantenbein, Thomas Gasser, Piotr Gawron, Lars Geffers, Virginie Giarmana, Enrico Glaab, Clarissa P. C. Gomes, Nikolai Goncharenko, Jérôme Graas, Mariela Graziano, Valentin Groues, Anne Grünewald, Wei Gu, Gaël Hammot, Anne-Marie Hanff, Linda Hansen, Maxime Hansen, Hulda Haraldsdöttir, Laurent Heirendt, Sylvia Herbrink, Sascha Herzinger, Michael Heymann, Karsten Hiller, Geraldine Hipp, Michele Hu, Laetitia Huiart, Alexander Hundt, Nadine Jacoby, Jacek Jarosław, Yohan Jaroz, Pierre Kolber, Joachim Kutzera, Zied Landoulsi, Catherine Larue, Roseline Lentz, Inga Liepelt, Robert Liszka, Laura Longhino, Victoria Lorentz, Clare Mackay, Walter Maetzler, Katrin Marcus, Guilherme Marques, Jan Martens, Conny Mathay, Piotr Matyjaszczyk, Patrick May, Francoise Meisch, Myriam Menster, Maura Minelli, Michel Mittelbronn, Brit Mollenhauer, Kathleen Mommaerts, Carlos Moreno, Friedrich Mühlschlegel, Romain Nati, Ulf Nehrbass, Sarah Nickels, Beatrice Nicolai, Jean-Paul Nicolay, Alberto Noronha, Wolfgang Oertel, Marek Ostaszewski, Sinthuja Pachchek, Claire Pauly, Magali Perquin, Dorothea Reiter, Isabel Rosety, Kirsten Rump, Venkata Satagopam, Marc Schlesser, Sabine Schmitz, Susanne Schmitz, Reinhard Schneider, Jens Schwamborn, Alexandra Schweicher, Janine Simons, Lara Stute, Christophe Trefois, Jean-Pierre Trezzi, Michel Vaillant, Daniel Vasco, Maharshi Vyas, Richard Wade-Martins, Paul Wilmes

**Affiliations:** 1 Luxembourg Centre for Systems Biomedicine (LCSB), University of Luxembourg, Campus Belval, Esch-sur-Alzette, Luxembourg; 2 School of Medicine, National University of Ireland, Galway, Ireland; 3 Department of Psychiatry and Psychotherapy, University Medicine Greifswald, Greifswald, Germany; 4 Integrated BioBank of Luxembourg, Dudelange, Luxembourg; 5 Parkinson Research Clinic, Centre Hospitalier de Luxembourg (CHL), Luxembourg City, Luxembourg; 6 Transversal Translational Medicine, Luxembourg Institute of Health (LIH), Strassen, Luxembourg; 7 Discipline of Microbiology, School of Natural Sciences, National University of Ireland, Galway, Ireland; 8 APC Microbiome, Cork, Ireland

**Keywords:** Parkinson’s disease, Gut microbiome, Computational modelling, Metabolic modelling, Transsulfuration pathway

## Abstract

**Background:**

Parkinson’s disease (PD) is a systemic disease clinically defined by the degeneration of dopaminergic neurons in the brain. While alterations in the gut microbiome composition have been reported in PD, their functional consequences remain unclear. Herein, we addressed this question by an analysis of stool samples from the Luxembourg Parkinson’s Study (*n* = 147 typical PD cases, *n* = 162 controls).

**Results:**

All individuals underwent detailed clinical assessment, including neurological examinations and neuropsychological tests followed by self-reporting questionnaires. Stool samples from these individuals were first analysed by 16S rRNA gene sequencing. Second, we predicted the potential secretion for 129 microbial metabolites through personalised metabolic modelling using the microbiome data and genome-scale metabolic reconstructions of human gut microbes. Our key results include the following. Eight genera and seven species changed significantly in their relative abundances between PD patients and healthy controls. PD-associated microbial patterns statistically depended on sex, age, BMI, and constipation. Particularly, the relative abundances of *Bilophila* and *Paraprevotella* were significantly associated with the Hoehn and Yahr staging after controlling for the disease duration. Furthermore, personalised metabolic modelling of the gut microbiomes revealed PD-associated metabolic patterns in the predicted secretion potential of nine microbial metabolites in PD, including increased methionine and cysteinylglycine. The predicted microbial pantothenic acid production potential was linked to the presence of specific non-motor symptoms.

**Conclusion:**

Our results suggest that PD-associated alterations of the gut microbiome can translate into substantial functional differences affecting host metabolism and disease phenotype.

## Background

Parkinson’s disease (PD) is a complex multifactorial disease, with both genetic and environmental factors contributing to the evolution and progression of the disease [1]. While several studies have elucidated the role of genetic factors in the pathogenesis of the disease [2–5], the role and the contribution of various environmental and lifestyle factors are still not completely understood [6]. Importantly, about 60% of the PD patients suffer from constipation [7], which can start up to 20 years before the diagnosis and is one of the prodromal syndromes [8, 9].

Human beings are considered to be superorganisms recognising the complex interplay between the host and microbes [10]. For instance, the human gut microbiome has been shown to complement the host with essential functions (trophic, metabolic, and protective) and to influence the host’s central nervous system (CNS) via the gut-brain axis through the modulation of neural pathways and GABAergic and serotoninergic signalling systems [11].

Recent studies have reported an altered gut composition in PD [12–20]. These studies have demonstrated that PD patients have an altered microbiome composition, compared to age-matched controls. However, the functional implications of the altered microbiome remain to be elucidated, e.g. using animal models [21]. A complementary approach is computational modelling, or constraint-based reconstruction and analyses (COBRA) [22], of microbiome-level metabolism. In this approach, metabolic reconstructions for hundreds of gut microbes [23] are combined based on microbiome data [24–26]. Flux balance analysis (FBA) [22] is then used to compute, e.g., possible metabolite uptake or secretion flux rates of each microbiome model (microbiome metabolic profile) [25] or to study microbial metabolic interactions (cross-feedings) [27, 28]. This approach has been applied to various microbiome datasets to gain functional insights [25, 26,29], including for PD where we have proposed that the microbial sulphur metabolism could contribute to observed changes in the blood metabolome of PD patients [29].

**Fig. 1:**
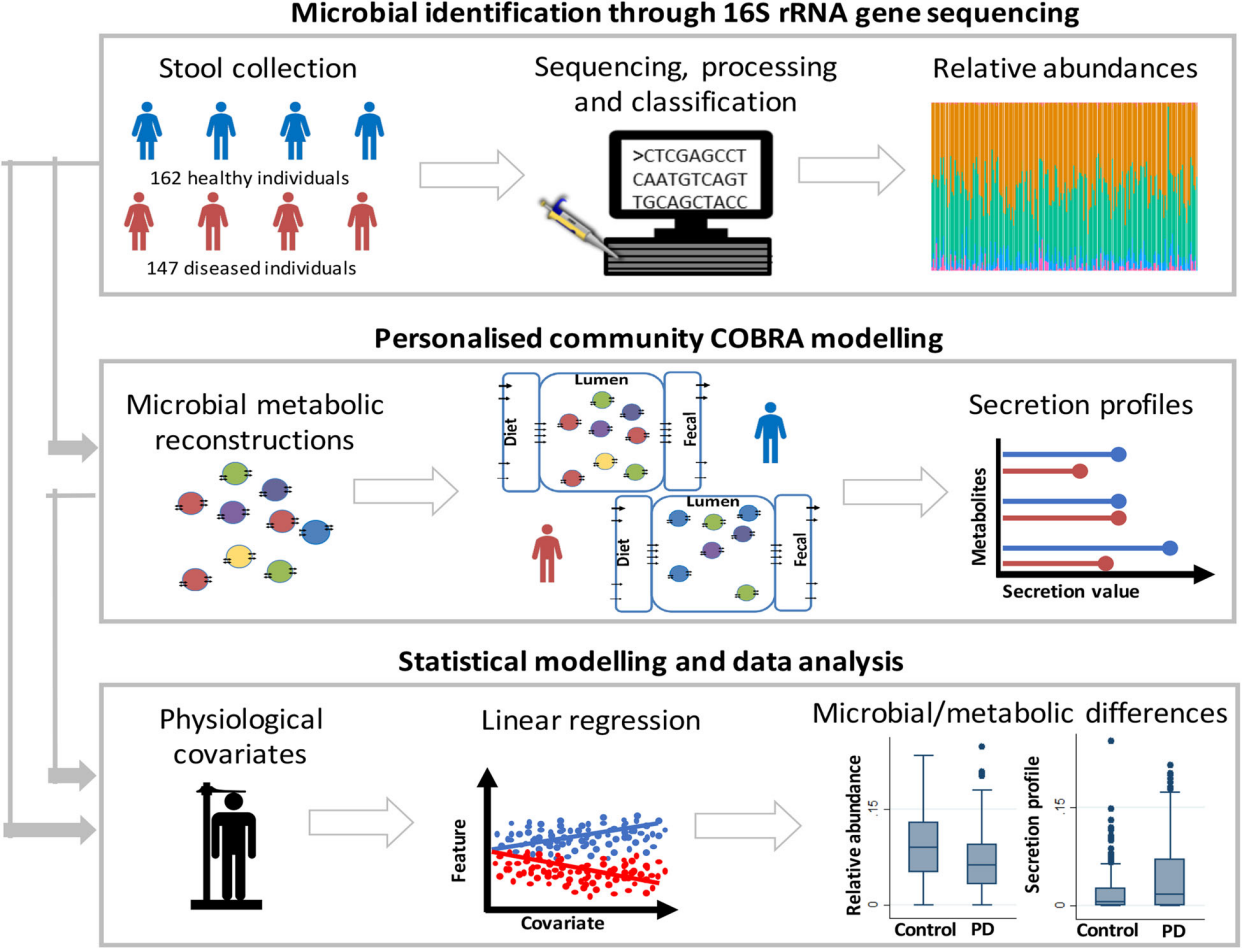
Overview of the study approach and the key methods used. Relative abundances were derived from 16S rRNA gene sequences (the “Analysis of the microbial composition with 16S rRNA gene sequencing” section) and used as an input for the personalised community modelling to simulate metabolite secretion profiles. Relative abundances and secretion profiles were statistically analysed to identify microbial or metabolic differences between PD patients and controls

In the present study, we aim at investigating microbial changes associated with PD while focusing on possible covariates influencing the microbial composition and at proposing functional, i.e. metabolic, consequences arising from the microbiome changes. First, we analysed the faecal microbial composition of PD patients and controls from the Luxembourg Parkinson’s Study [30] (Fig. 1). Second, based on the observed significant differences in the composition of microbial communities between PD patients and controls, we created and interrogated personalised computational models representing the metabolism of each individual’s microbial community. We demonstrate that the combined microbial composition and functional metabolite analysis provides novel hypotheses on microbial changes associated with PD and disease severity, enabling future mechanism-based experiments.

## Results

The Luxembourg Parkinson’s Disease study includes patients with typical PD, atypical parkinsonism, and secondary parkinsonism of any disease stage as well as age/sex-matched healthy control subjects from Luxembourg and its neighbouring regions from a broad age range [30]. For the present study, we focused on typical PD patients and healthy controls over the age of 50 (Table 1, the “Methods” section). Stool samples were analysed for 147 PD patients and 162 controls using 16S rRNA gene sequences (the “Analysis of the microbial composition with 16S rRNA gene sequencing” section). From these 309 individuals, one individual had to be excluded from analyses because of missing body mass index (BMI), resulting in 308 individuals that were included in statistical analyses. Note that the case numbers for individual statistical analyses may be lower because of missing values in other variables (e.g. clinical assessment) (see Table 1).

**Table 1:** Descriptive statistics of the analyses sample from the Luxembourg Parkinson’s Disease Study

Variable	PD	Control	Missing values	
			PD (%)	Control (%)
Cases vs. controls	147	162	0	0
Female subjects	31.5%	35.8%	0	0
Age at basic assessment (mean ± SD)	69.3 ± 8.6	63.3 ± 8.3	0	0
Body mass index (mean ± SD)	27.3 ± 4.5	27.9 ± 4.8	0.7	0
Sniff score (mean ± SD)	7.1 ± 3.4	12.7 ± 2.1	0	0
Diabetes	4.1%	3.1%	0	0
Non-motor symptoms questionnaire score (NMS-PD) (mean ± SD)	9.3 ± 5.1	3.9 ± 3.9	9.5	3.7
Constipation	36.7%	6.2%	0	0
PD disease duration since diagnosis	5.9 ± 5.7	–	6.1	–
UPDRS-part I (mean ± SD)	10.0 ± 5.9	4.5 ± 4.4	3.4	3.1
UPDRS-part II (mean ± SD)	11.8 ± 8.1	1.3 ± 2.8	1.4	2.4
UPDRS-part III (mean ± SD)	34.6 ± 16.1	2.3 ± 2.9	1.4	0
UPDRS-part IV (mean ± SD)	1.7 ± 3.2	–	1.4	–
Hoehn and Yahr (mean ± SD)	2.2 ± 0.6	–	0	–
L-DOPA intake	66.7%	0%	0	0
Dopamine agonist intake	56.5%	0%	0	0
MAO-B inhibitor intake	41.5%	0%	0	0
COMT inhibitor intake	4.1%	0%	0	0

PD disease duration refers to the time since diagnosis at the date of stool sampling

*SD* standard deviation, UPDRS Unified Parkinson Disease Rating Scale, *L-DOPA* levodopa, *MAO-B* monoaminooxidase B, *COMT* catecholamine-methyl-transferase, *NMPC* net maximal production capability, “–” no value to report

### Beta diversity is altered in PD microbial communities

We analysed alpha and beta diversity indices across healthy controls and PD microbiomes. These analyses were carried out on the 308 individuals with complete covariate data via linear regressions. In terms of alpha diversity, we calculated the richness in species, Shannon entropy, and the evenness Pielou indices [31]. The Shannon entropy did not significantly differ between PD cases and controls, in agreement with earlier studies [12, 15, 20] but in disagreement with two different PD studies [13, 16]. However, the species richness was slightly increased in PD (regression coefficient *b* = 4.76, 95% confidence interval (CI) 0.44;9.08, *p* = 0.03) (Additional file 1: Fig. S1). Importantly, we found a significant sex-group interaction term regarding the Pielou index with the effect sign of PD being reversed. The Pielou index was reduced in female PD patients but increased in male PD patients (Additional file 1: Fig. S1). Most noticeable, however, was the increased variance in the Pielou index in men (Additional file 1: Fig. S1). These results indicate a sex dependence of the alpha diversity, although this isolated result needs validation. However, sex-dependent microbiome changes in the context of disease were at least described in a mouse model of inflammatory bowel disease and are discussed in the context of the gut-brain axis [32, 33]. Thus, sex-microbiome interaction in health and disease deserves further investigation.

In terms of beta diversity, the performed ANOSIM analyses of the Bray-Curtis dissimilarities indicated small but significant differences between PD and healthy microbial communities (ANOSIM statistics *R* = 0.04, *p* = 0.001). Thus, beta diversity, corroborating earlier results [12, 13, 15–18, 20, 34–36]), differs between healthy and PD microbial communities (Additional file 1: Fig. S2).

### Species and genus level changes in PD microbiomes

**Fig. 2:**
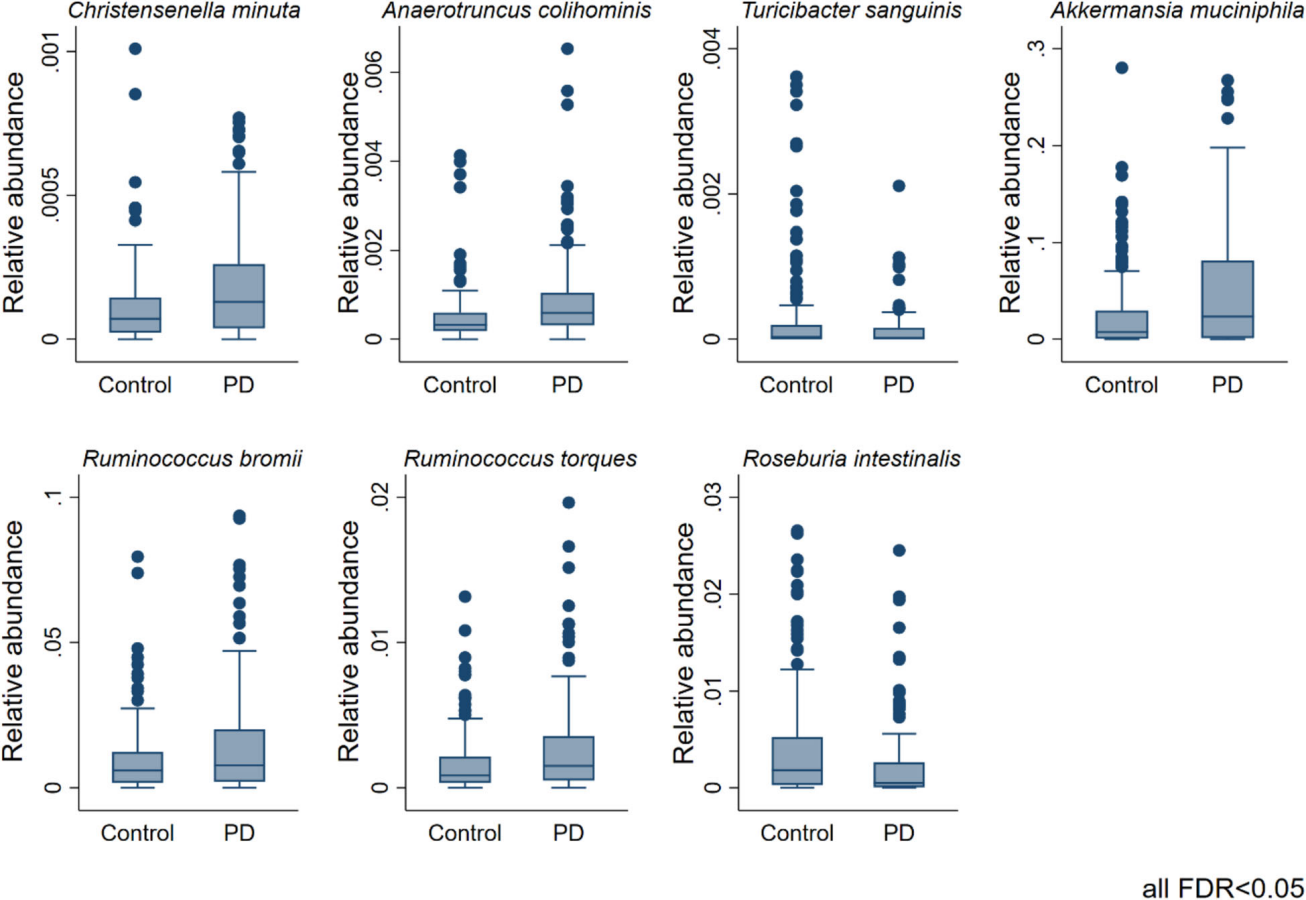
Boxplots of seven significantly changed species in PD vs. controls (FDR < 0.05). Significance levels were determined using multivariable semi-parametrical fractional regressions with the group variable (PD vs. control) as a predictor of interest, including age, gender, BMI, and technical variables (total read counts and sequencing run (batch)) as covariates. FDR, false discovery rate

**Fig. 3:**
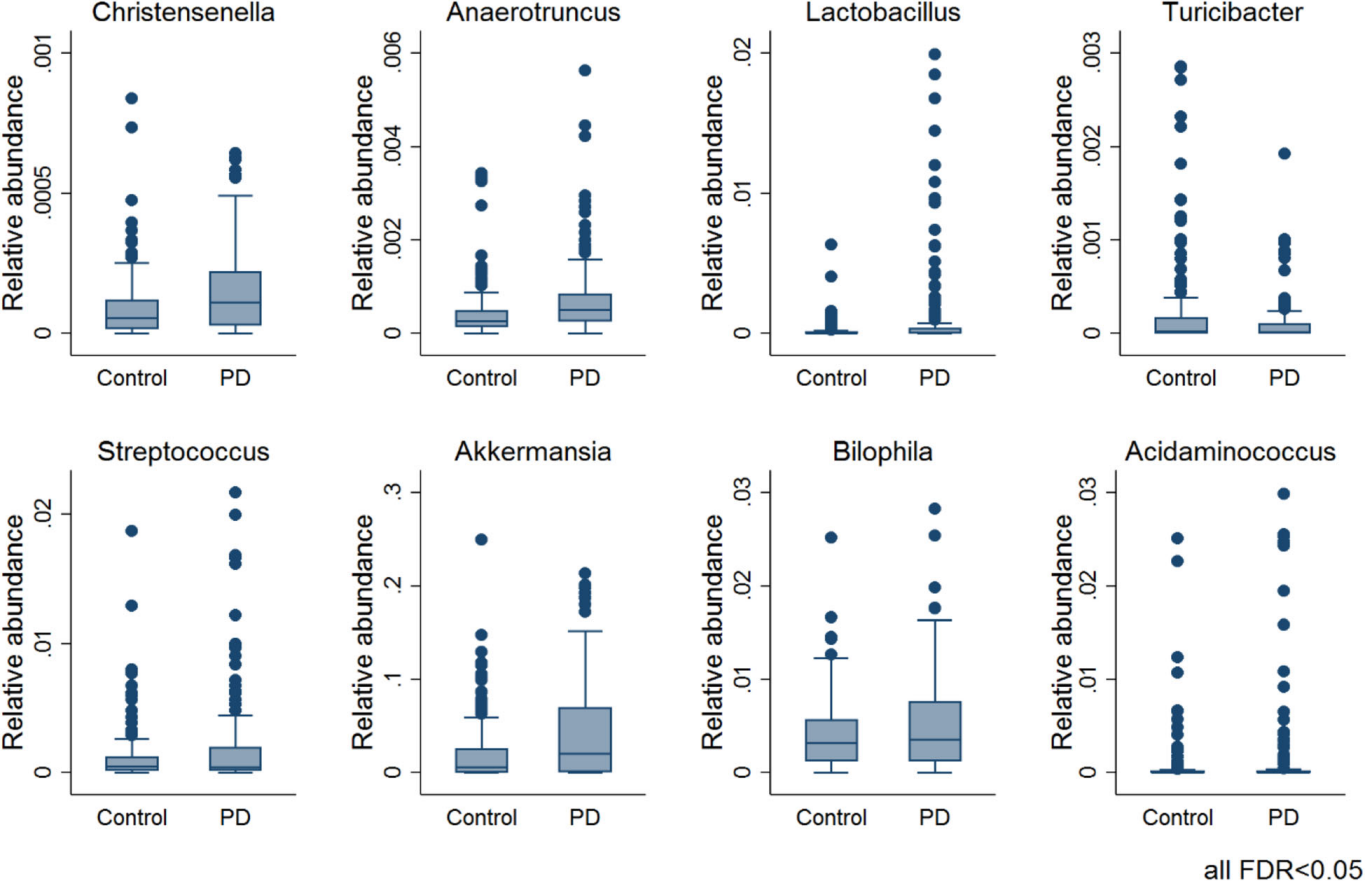
Boxplots of eight significantly changed genera in PD vs. controls (FDR < 0.05). Significance levels were determined using multivariable semi-parametrical fractional regressions with the group variable (PD vs. control) as a predictor of interest, including age, gender, BMI, and technical variables (total read counts and sequencing run (batch)) as covariates. FDR, false discovery rate

We investigated disease-associated microbial changes at the species level. Seven species were significantly altered in PD (FDR < 0.05, Fig. 2) in multivariable fractional regressions on the 308 individuals with complete covariate data. Note that when comparing results between different taxonomic levels, changes observed for *Ruminococcus* and *Roseburia* species were not significant on the genus level but on the species level, highlighting the importance of species-level resolution. The highest effect size on the species level was associated with *Akkermansia muciniphila* (odds ratio (OR) = 1.80, 95% CI = (1.29, 2.51), *p* = 6.02e−04, FDR < 0.05) in agreement with the previously reported higher abundance of *A. muciniphila* in PD patients [12, 13]). This odds ratio of 1.8 means that the odds of a certain sequence read being assigned to *A. muciniphila* was estimated to be 80% higher in PD cases than in controls. These odds ratios and later estimates were calculated from the fractional data (e.g. the relative abundance data). Subsequently, we examined possible differences at the genus level by performing semiparametric fractional regressions while adjusting for age, sex, the body mass index (BMI), batch, and total read counts. We identified eight genera to be significantly increased in PD (FDR < 0.05; Fig. 3, Table 2), with *Lactobacillus* showing the highest effect size (odds ratio (OR) = 5.75, 95% CI = (2.29, 14.45), *p* = 1.96e−04, FDR < 0.05, Fig. 3). In contrast, the genera *Turicibacter* decreased significantly in PD cases (FDR < 0.05). We repeated these analyses adjusting additionally for constipation to account for this potential confounder in sensitivity analyses. All genera and species remained significant except for the *Ruminococcus* species. To summarise, significant changes could be observed on the species and genus levels.

**Table 2:**
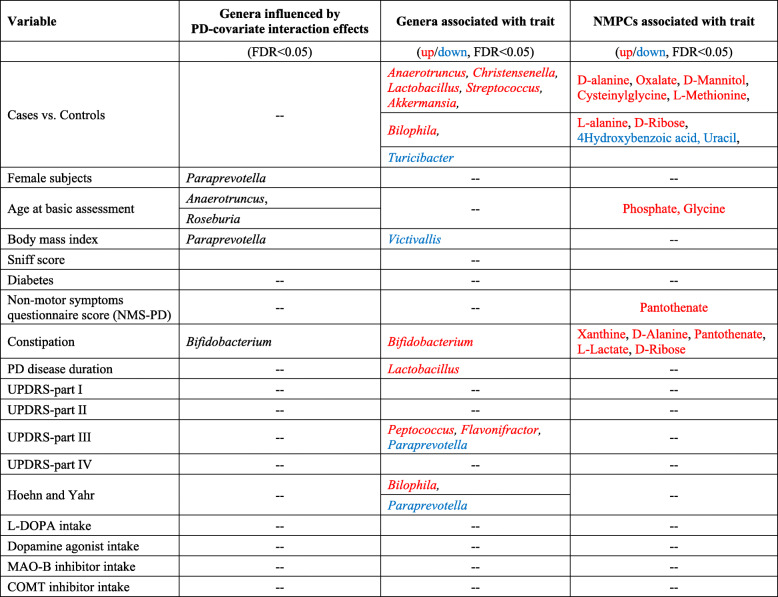
Disease study and overview of associations

Red label means a significant positive association (FDR < 0.05) with the variable denoted in the “variable” column, blue label means a significant negative association, and “–” means no significant result after correction for multiple testing. PD disease duration refers to the time since diagnosis at the date of stool sampling

*UPDRS* Unified Parkinson Rating Scale, *L-DOPA* levodopa, *MAO-B* monoaminooxidase B, *COMT* catecholamine-methyl-transferase, *NMPC* net maximal production capability

### PD modifies the effects of basic covariates on the microbiome

**Fig. 4:**
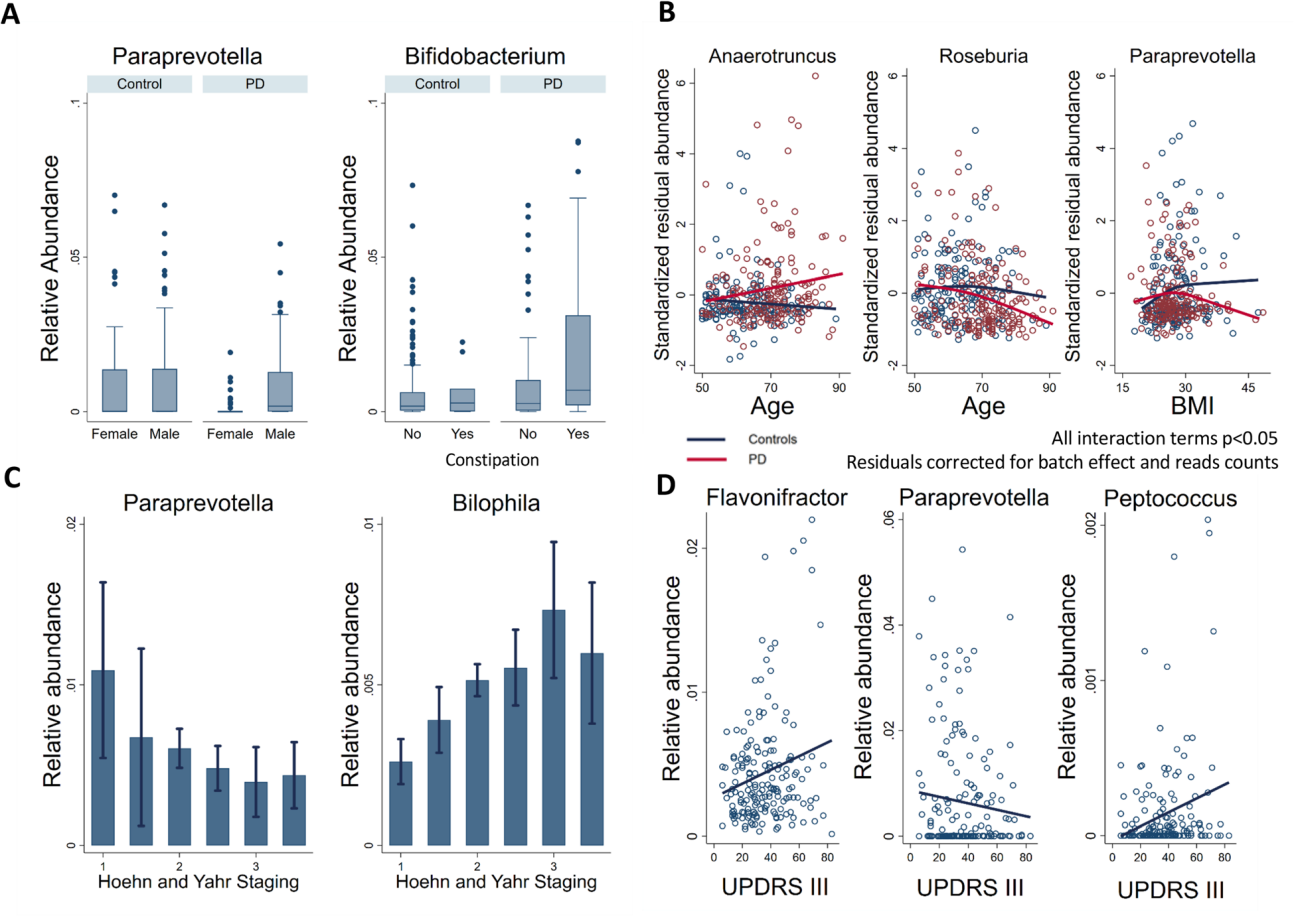
Genus alterations in PDs in the context of basic covariates and clinical variables. **a** Boxplots of *Paraprevotella* and *Bifidobacterium* for cases and controls in dependence of sex and constipation, respectively. In both cases, differences in mean abundance had FDR < 0.05. **b** Scatter plots and non-linear regression lines for cases and controls of genus abundances of *Anaerotruncus*, *Roseburia*, and *Paraprevotella* in dependence of age and BMI. Global test (Wald test, testing all interaction terms simultaneously on zero) had an FDR < 0.05 in all three cases. For graphical assessment of the interaction terms, the *z*-transformed residual abundances are displayed after correction for technical covariates (batch and read counts). **c** Error bar plots of *Paraprevotella* and *Bilophila* abundances in dependence of disease staging. Genus association with disease staging showed a decrease of relative abundance of *Paraprevotella* and an increase of *Bilophila* genus over increasing Hoehn and Yahr scale values (FDR < 0.05). Error bars represent 95% confidence intervals. **d** Scatter plots of motor symptoms (UPDRS-part III) were positively associated with of *Flavonifractor* and *Peptococcus* abundances and negatively with *Paraprevotella* abundance (FDR < 0.05). UPDRS, Unified Parkinson Rating Scale; BMI, body mass index; FDR, false discovery rate

Furthermore, we investigated whether the genus-level alterations in PD were affected by basic confounding factors using multivariable fractional regressions based on the data from 308 individuals with complete covariate data. This interaction analyses uncovered rich effect modifications, revealing that microbiome changes in PD should be considered in the context of age, BMI, and gender. Our analyses demonstrated that the effects of PD were not homogeneous amongst important subgroups of patients. For example, *Paraprevotella* was exclusively reduced in female patients in comparison with male participants but not in female controls (Fig. 4a), highlighting gender-dependent alterations of microbial communities in PD. In addition, the effects of BMI and age were modified in PD cases. The PD cases had increased *Anaerotruncus* abundance with age, while non-linear, overall decreasing abundances of *Roseburia* and *Paraprevotella* were observed with age and BMI, respectively (Fig. 4b). Taken together, these analyses suggest that microbial abundances are shifted in PD cases and that also the effects of important covariates were altered in PD, reflecting the systemic and complex nature of PD.

### Microbial abundances, medication intake, and constipation in PD

The Luxembourg Parkinson’s Study has enrolled patients of all stages of PD [30]. Therefore, the patients have considerable inter-individual variance in PD-related features, such as constipation and intake of medication (Table 1). We analysed whether these features had an impact on the microbiome composition in PD via multivariable fractional regressions on the data of all 308 study participants with complete covariate data. In our data, we could not find any evidence for an effect of the three medication types on the microbiome, i.e. levodopa, or MAO-B inhibitors, when correcting for multiple testing (Additional file 1: Table S2). Noteworthy, we were not able to investigate the effects of COMT inhibitors due to the small number of cases (*n* = 6). In contrast, constipation, a prevalent non-motor symptom in PD patients [37], was associated with an increased abundance of *Bifidobacterium*, with a clear effect in constipated PD cases (Fig. 4a). However, since there were only ten constipated controls (Table 2), these results must be confirmed in larger cohorts.

### Genus association with the disease severity

We next investigated whether the stage of the disease, i.e. defined by Hoehn and Yahr staging, non-motor symptoms scale (NMS-PD), and Movement Disorder Society-Unified Parkinson’s Disease Rating Scale (MDS-UPDRS; further abbreviated as UPDRS) scores and its subscales, was associated with altered genus abundance. Because of missing data, the case numbers included into the statistical analyses varied between those variables with *n* = 146 in case of Hoehn and Yahr staging, *n* = 133 for the NMS-PD, *n* = 145 for the UPDRS scales, and *n* = 138 for the disease duration. For the Hoehn and Yahr staging, *Paraprevotella* showed a negative association and *Bilophila* showed a positive association, both of which were significant after multiple testing (Fig. 4c). For the UPDRS III subscale score (i.e. motor symptoms, Table 2), three genera, being *Peptococcus*, *Flavonifractor*, and *Paraprevotella*, survived correction for multiple testing (Fig. 4d). In contrast, the other UPDRS subscales and the NMS-PD were not significantly associated with microbial changes, after correction for multiple testing. Note that these analyses were performed while adjusting for disease duration. When analysing the association pattern of disease duration, we found *Lactobacillus* positively correlated with the disease duration (FDR < 0.05, Additional file 1: Fig. S3). In conclusion, our data suggest that the microbial composition may be utilised as a correlate of disease severity.

### Metabolic modelling reveals distinct metabolic secretion capabilities of PD microbiomes

**Fig. 5:**
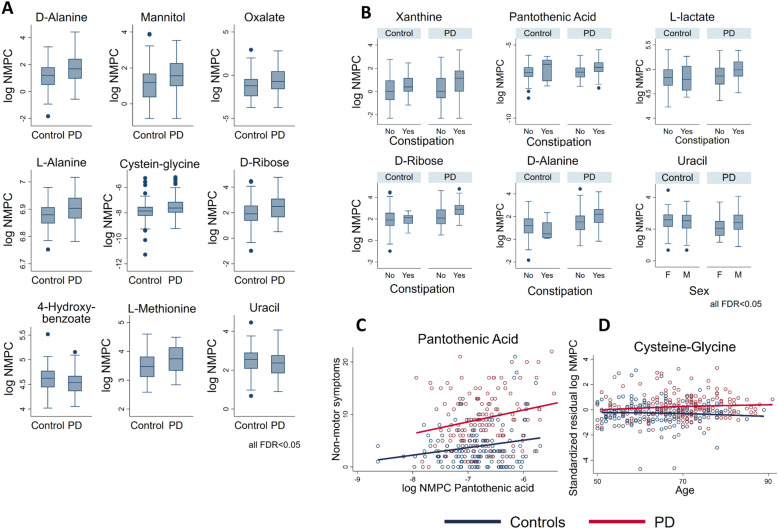
Overview of the significantly different predicted net metabolite production potentials (NMPCs) of microbial communities from PD patients and healthy individuals. **a** Box plots for NMPC differential between cases and controls with FDR < 0.05. **b** Box plots for NMPCs with sex-specific PD signature or constipation effects (all FDR < 0.05). **c** Scatter plot of non-motor symptom scores and the NMPC of pantothenic acid displaying the regression lines for cases and controls. The slope was significantly bigger than zero (FDR < 0.05). **d** Scatter plot of cysteine-glycine NMPC and age for cases and controls displaying the corresponding regression lines. The difference in slopes of regression lines was significant (FDR < 0.05). FDR, false discovery rate

To obtain insight into the possible functional consequence of observed microbiome changes in PD, we used metabolic modelling (cf. the “Methods” section and Additional file 1). Briefly, we mapped each of the 308 microbiome samples with complete covariate data on the generic microbial community model consisting of 819 gut microbial reconstructions [23, 25] to derive personalised microbiome models [24]. We then computed the net maximal production capability (NMPC), or maximal secretion flux potential, for 129 different metabolites that could be secreted by each microbial community model (cf. “Methods” section), providing thereby a characterisation of the differential microbial metabolic capabilities in PDs and controls. For one individual, the computation failed as the applied diet constraints resulted in an infeasible community model. Consequently, the statistical analyses, via multivariable mixed effect linear regression, were performed on computational modelling results of 307 community models. The predicted NMPCs of nine metabolites were different in PD (Fig. 5a, all FDR < 0.05). Moreover, although less dominant in comparison with the abundance data, PD-covariate interactions were also prevalent, with the predicted uracil NMPC showing a sex-specific effect and cysteine-glycine showing a age-dependent PD effect (Fig. 5b, d). In subsequent analyses, we tested for associations of the NMPCs with constipation, medication, disease duration, Hoehn-Yahr staging, NMS, and UPDRS III scores, complementing thereby the analyses on the abundance level. Notably, we found the NMPCs of xanthine, d-alanine, l-lactic acid, d-ribose, and pantothenic acid positively associated with constipation (Fig. 5b), while no NMPC was associated with medication or with disease duration. However, the NMPC of pantothenic acid was positively associated with higher NMS scores, interestingly both in PD and in controls (Fig. 5c). No NMPC survived correction for multiple testing regarding associations with the UPDRS III score and Hoehn-Yahr staging. To conclude, these results suggest that the altered microbial composition in PD could result in broad changes in metabolic capabilities, which manifested themselves additionally in non-motor symptoms and constipation.

### PD-specific secretion profiles were altered due to changed community structure and species abundances

**Fig. 6:**
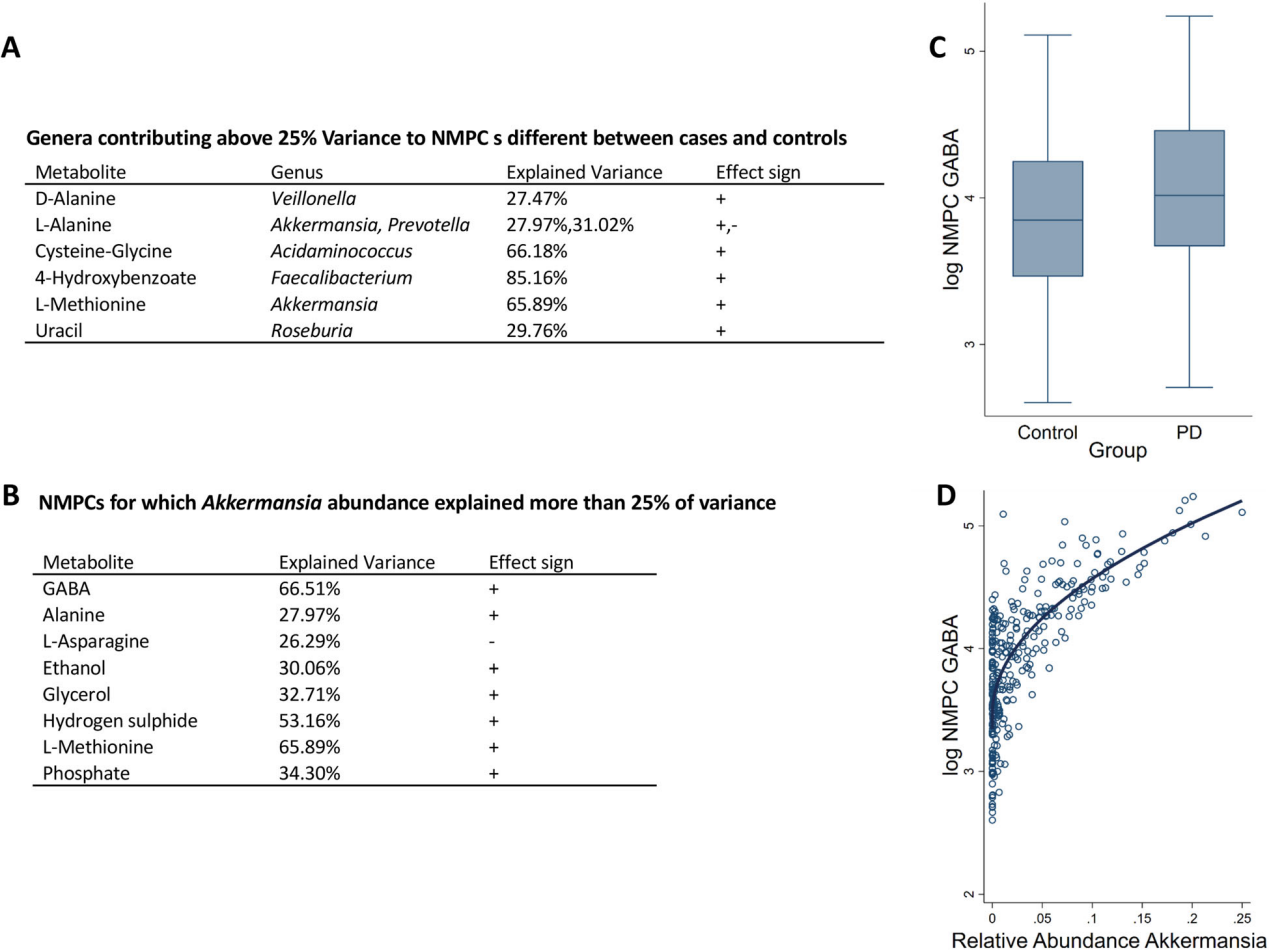
Overview of the analyses of species contribution to NMPCs. **a** Table of genera explaining more than 25% of the variance in metabolite NMPCs different between cases and controls. **b** Table of NMPCs, for which *Akkermansia* explained at least 25% of the variance in the total to community net production capacity. **c** Box plots of gamma-aminobutyrate (GABA) net production capacity for PD cases and controls (*b* = 0.18, 95% CI 0.06, 0.30, *p* = 0.003, FDR = 0.0501). **d** Scatter plot of GABA net production capacity in dependence of Akkermansia abundance with non-linear regression lines. NMPC, = net maximal production capacity; GABA, gamma-aminobutyrate; FDR, false discovery rate. Effect sign “–”: negative correlation. Effect sign “+”: positive correlation

Next, we analysed which microbes contributed to the predicted differential NMPCs by correlating them to the abundance data (Fig. 6). These analyses were performed via linear regressions on the 307 cases with complete covariate data and feasible community models. For six NMPCs, large portions of the observed variance could be explained by single genus (Fig. 6a), while for the other four NMPCs, no single dominant genus could be identified. In addition, we computed the variance explained by each genus for the predicted NMPCs of each secreted metabolite. From the PD-associated genera, only *Akkermansia*, *Acidaminococcus*, and *Roseburia* explained over 25% of the variances in NMPCs. *Acidaminococcus* was responsible for 64% of the variance in cysteine-glycine production and *Roseburia* for 30% of the variance in uracil production potential. *Akkermansia* impacted the predicted NMPCs the most by substantially explaining variances in the predicted NMPCs of nine metabolites (Fig. 6b), including the neurotransmitter gamma-aminobutyric acid (GABA, Fig. 6d) and two sulphur species, being hydrogen sulphide and methionine. GABA was also significantly altered between PD and controls on a nominal level, missing FDR-corrected significance narrowly (*b* = 0.18, 95% CI 0.06;0.30, *p* = 0.003, FDR = 0.0501, Fig. 6c). Note that the relation between microbial abundance and NMPC is not necessarily linear as shown in the case of GABA and *Akkermansia* (Fig. 6d). These analyses demonstrate the added value of metabolic modelling to investigate altered metabolic functions of the whole microbial composition.

## Discussion

In this study, we aimed at elucidating compositional and functional changes in the faecal microbiome of PD patients. Therefore, we analysed 16S rRNA gene sequencing data from a cohort of typical PD patients (*n* = 147) and controls (*n* = 162) and performed personalised microbial computational modelling. We identified (i) eight genera and seven species that changed significantly in their relative abundances between PD patients and healthy controls; (ii) PD-associated microbial patterns that were dependent on sex, age, BMI, and constipation; and (iii) in PD patients, altered metabolite secretion potentials, predicted using metabolic modelling of microbial communities, were found particularly for sulphur metabolism. Overall, our work demonstrated compositional and predicted functional differences in the gut microbial communities of Parkinson’s disease patients providing novel experimentally testable hypothesis related to PD pathogenesis.

**Fig. 7:**
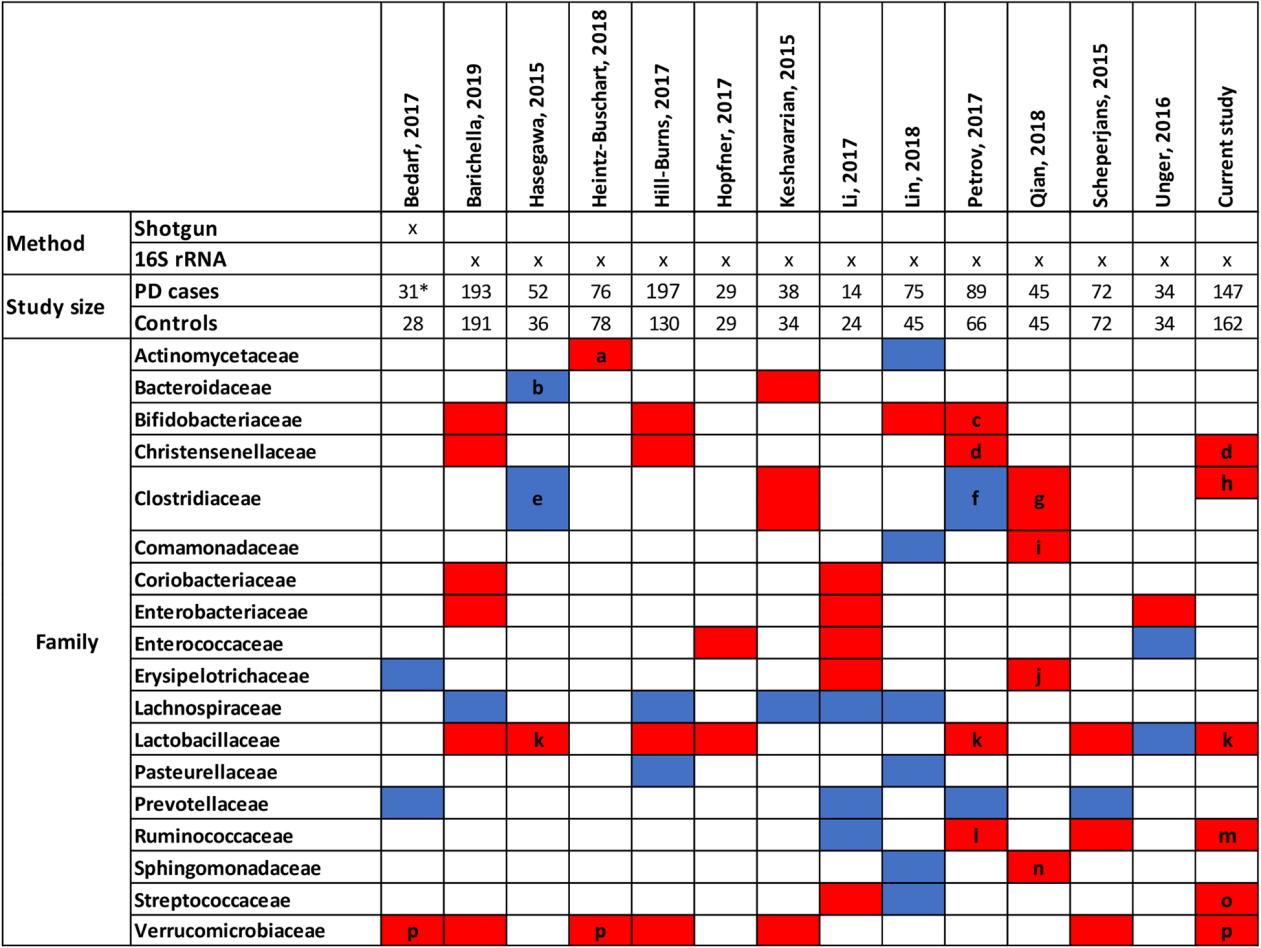
Reported microbial changes at the family level associated with PD in different studies. Only those bacterial families are shown, for which significant associations with species or genera have been reported in at least two studies comparing stool samples from patients and controls. Red—increased in PD; blue—decreased in PD. a: *Actinomycetales*; b: *Bacteroides fragilis*; c: *Bifidobacterium*; d: *Christensenella*; e: *Clostridium coccoides*/*leptum*; f: *Faecalibacterium* and *Dorea*; g: *Clostridium* IV/XVIII, *Butyricicoccus* and *Anaerotruncus*; h: *Anaerotruncus*; i: *Aquabacterium*; j: *Holdemania*; k: *Lactobacillus*; l: *Oscillospira*; m: *Ruminococcus romii* and *Ruminococcus torques*; n: *Sphingomonas*; o: *Streptococcus*; p: *Akkermansia*. *Drug-naive, de novo PD patients only, Based on [14]

The microbial compositional analyses of our cohort identified significantly different microbial abundance distributions between PD patients and healthy controls (Table 2). An increasing number of studies have described altered colonic microbial compositions associated with PD, and an overall picture starts to arise [38] (Fig. 7). For instance, the microbial families of *Verrucomicrobiaceae* and *Lactobacillaceae* have been consistently found to have an increased abundance in PD (Fig. 7). In accordance, our study also reports increased abundance in PD of *Akkermansia*, *Christensenella*, and *Lactobacillus*. Similarly, *Bifidobacteria* has also been repeatedly associated with PD (Fig. 7), but in our study, we could show that the *Bifidobacteria* association was dependent on constipation (Fig. 4a) highlighting the need for incorporating disease-specific phenotypes as covariates into the statistical design.

At the same time, inconsistencies between the studies remain, and they may be due to the differences in the study design, inclusion criteria, faecal sampling, the use of whole-genome sequencing [12], 16S marker gene regions to be targeted, DNA extraction protocols, and statistical methods. For instance, we used a relatively large, PD cohort while Bedarf and colleagues [12] studied a small, well-defined cohort of drug-naive, male PD patients and male controls (Fig. 7). Three studies included individuals of Chinese descent [34–36], one conducted with qPCR assay which included individuals from Japan [19], while the other studies focused on Caucasian individuals. It has been shown that microbial composition is associated with ethnic background, geography, and dietary habits [39–41], which may explain some of the discrepancies. The differences between the studies, hence, highlight the importance of performing a meta-analysis to identify global microbial signatures, as it has been done for, e.g., colorectal cancer [42]. Such meta-analysis may also permit to investigate subgroups of PD, as the number of cases and controls would be substantially increased and thus provide higher statistical power. For instance, we observed various effect modulators that were not reported before in humans (Table 2), such as *Paraprevotella* abundance reduction being specific to women. This result is apparently in contradiction with the findings from Bedarf and colleagues [12] who reported decreased levels of *Prevotellaceae* in a cohort of only male PD patients. However, since alterations on the genus level may compensate for the ones detected at a family taxonomic level (e.g. lowered *Paraprevotella* but higher *Prevotella*), the results described on the genus level are not directly comparable to the results on the phylum level. Additionally, and as highlighted above, differences might be due to sex-specific effects, as observed here (Fig. 4a). Accordingly, a study reported a higher abundance of *Paraprevotella* in male mice compared to female mice [43]. Despite the lack of extensive studies on gender-specific differences in microbiome composition, we suggest that machine learning procedures on microbiome data should be performed in a sex-stratified manner. Larger cohorts, e.g. through a meta-analysis of published cohorts, would allow the identification of generalisable microbial differences in PD patients and also specific microbial changes associated with certain traits and physiological characteristics, as suggested by our data.

We could not detect any evidence for an effect of the dopaminergic, PD-specific medication on the microbiome composition, after correction for multiple testing. It should be noted that, with our study design, we cannot rule out impacts of dopaminergic medication on the microbiome. To detect small effect sizes of dopaminergic medication, a higher sample size would be required than present in our cohort. It is also to be noted that PD medication is often taken in conjunction with other drugs, again requiring larger sample sizes, than used in our study, to permit the investigation of all possible drug combinations. For instance, the potential effects of COMT inhibitors could not be analysed in this study because of missing sample size. Nonetheless, in previous studies, *Dorea* and *Phascolarctobacterium* genera have been negatively associated with levodopa equivalent doses [36], and members of the family of *Bacillaceae* have been correlated with levodopa treatment [13]. It is also to be considered that levodopa is absorbed in the upper part of the small intestine [44], and thus, small intestinal rather than large intestinal microbes may play a more prominent role in levodopa bioavailability. Consistently, a recent study showed that bacterial tyrosine decarboxylases restrict the bioavailability of levodopa [45]. Interestingly, 193 of the 818 (24%) gut microbes with genome-scale metabolic reconstructions [23, 46] carry the necessary genes encoding for proteins that convert levodopa into dopamine. Levodopa is always given with decarboxylase inhibitors, such as carbidopa or benserazide, targeting the human decarboxylases, but it cannot be excluded that they also act on the microbial counterpart. However, van Kessel et al. have shown that carbidopa and benserazide are only a weak inhibitor of the microbial tyrosine decarboxylase [45].

We identified a positive association of *Bilophila* abundance with the Hoehn and Yahr staging, which captures motor impairment and disability independent of disease duration. Indeed, the abundance of *Bilophila* was not associated with disease duration indicating mainly the dependency on the progression of symptoms. This finding is consistent with experimental mouse studies demonstrating the pro-inflammatory effect of *Bilophila* overgrowth [47, 48]. *Bilophila* has a unique capability amongst the microbes covered by AGORA [23] to use taurine, an inhibitory neurotransmitter with neuroprotective effects [49, 50], as an energy source [51]. This pathway involves the pyruvate aminotransferase [51], which converts pyruvate and taurine into l-alanine and sulfoacetaldehyde, respectively. As *Bilophila* was significantly increased in PD cases (FDR < 0.05) and *Bilophila* represents the only genus capable of using taurine for ATP generation, PD microbiomes are in consequence enriched for this specific metabolic function (generation of ATP from taurine). In a previous study [29], we have shown that blood taurine-conjugated bile acids were positively associated with motor symptoms. We have proposed that *Bilophila* may be a marker of disease progression in PD, and it could modulate human sulphur metabolism through its taurine degradation capabilities [29]. Accordingly, we have reported alterations in sulphur metabolism when using computational modelling of microbiomes [29] from a cohort of early diagnosed and levodopa-naive PD patients [12] as well as an increased concentration of methionine and derived metabolites in blood samples [29]. Furthermore, we and others have reported alterations in bile acids and taurine-conjugated bile acids in PD patients [29, 52]. In accordance, our present study found *Bilophila* to be associated with disease severity strengthening the link between *Bilophila*, taurine, and Parkinson’s disease.

Interestingly, an increased abundance of *Bilophila wadsworthia* has been linked to constipation [53], which another study on individuals with chronic constipation has reported a decrease in *Bifidobacteria* abundance [54]. We found an increase in *Bifidobacteria* abundance in constipated individuals and, particularly, in constipated PD patients; however, the number of constipated controls in our study was very low (*n* = 10). In contrast, we could not find statistically significant changes in the association between the abundance of *B. wadsworthia* and individual constipated PD patients (Fig. 4c). Overall, the available data suggest that complex alterations in the microbial composition are associated with constipation but may differ between diseases. Hence, whether *B. wadsworthia* plays a role in constipation of PD patients needs to be further investigated.

The mucin-degrading microbe, *A. muciniphila*, represents about 1–4% of the faecal microbiome in humans [55]. Numerous diseases have been associated with a decrease in *A. muciniphila* abundance [56, 57], while an increase has been consistently reported in PD patients (Fig. 7). The *A. muciniphila* abundance had the largest contribution to the significantly altered metabolite secretion profiles (Fig. 6b), including the neurotransmitter gamma-aminobutyric acid (GABA). While its predicted secretion potential was only nominally increased in PD patients in the present study, higher GABA secretion rates have also been predicted based on the microbiome data from early-stage levodopa-naive PD patients [29]. Importantly, GABA receptors have been found in the enteric nervous system, gut muscle, gut epithelial layers, and endocrine-like cells [58], and its gut receptors are thought to be related to gastric motility (peristalsis), gastric emptying, and acid secretion [58]. Experiments with the GABA_b_ receptor agonist baclofen have shown that GABA_b_ receptors can reduce gastric mobility in the colon of rabbits via cholinergic modulation [59]. GABA could reach the CNS via the bloodstream as a lipophilic compound, being able to pass the blood-brain barrier. Additionally, microbial GABA could affect the brain-gut axis by contributing the human GABA pools, especially as it has been shown that the microbiome can affect GABA receptor density in the CNS via the vagus nerve [60]. Interestingly, *A. muciniphila* has been shown to be positively associated with gastrointestinal transit time [61, 62], so one may hypothesise that this effect may be due to its GABA production capability. To establish whether and which role *A. muciniphila* and GABA may play a role in prodromal PD, further experimental studies will be required.

In order to move beyond mere cataloguing of microbial changes associated with diseases, pathway-based tools (e.g. [63]) have been developed, in which microbial sequences (or reads) are mapped, e.g. onto the Kyoto Encyclopedia of Genes and Genomes (KEGG) ontologies present in the KEGG database [64]. Using such tools, Bedarf et al. reported decreased glucuronate degradation and an increase in tryptophan degradation and formate conversion [12]. Similarly, Heinz-Buschart et al. reported 26 KEGG pathways to be altered in PD microbiomes [13]. In our study, we complemented the compositional analysis with computational modelling to gain insight into potential functional, i.e. metabolic, consequences of changed microbe abundances in PD. The advantage of our approach is that the functional assignments may be more comprehensive than more canonical methods, such as KEGG ontologies, because (1) the underlying genome-scale metabolic reconstructions have been assembled based on refined genome annotations and have been manually curated to ensure that the reaction and gene content is consistent with current knowledge about the microbe’s physiology [65], and (2) each of these reconstructions, alone or in combinations, is amenable to metabolic modelling, and thus, functional and metabolic consequences of a changed environment (e.g. nutrients or other microbes in the models) can be computed [22]. These simulations are thus allowing to predict functional consequences and not only pathway or reaction enrichment, as typically done.

### Strengths and limitations

Here, we presented microbiome analyses in a large monocentric longitudinal study on PD (including cases and controls) with a nation-wide outreach in Luxembourg and the adjacent border regions (Greater Region), which includes an clinical spectrum of all disease stages [30]. We demonstrated that the microbial composition is not only altered in PD but also that the observed associations of PD with changes in the composition of the microbiome should be interpreted in the context of age, sex, BMI, and constipation. This information is of importance for clinical translation, highlighting the need for both (i) a personalised and (ii) a holistic approach, in order to understand the role of microbial communities in PD pathogenesis. In a second step targeting the potential functional changes related to PD-associated microbiomes, we performed metabolic modelling based on the AGORA collection [23] of genome-scale metabolic reconstructions, allowing for the predictions of metabolite secretion profiles. Thus, our analyses facilitated a detailed investigation of the altered metabolism of PD-related microbial communities in the gut pointing towards a role of the known pro-inflammatory species *B. wadsworthia* interacting with the host on sulphur metabolism. Hence, metabolic modelling provides a valuable tool for deciphering the metabolic activity of microbial communities in PD.

However, despite the partial confirmation of previous results by our study (Fig. 7), several limitations should be kept in mind. First, certain covariates were not investigated, such as diet, exercise, and smoking. Whether these covariates alter the PD-specific signature has yet to be analysed. Although our study belongs to the three largest studies performed on PD, our sample size was still too small to deliver insights on combinations of drugs, as the statistical power to detect effects of drugs was lower than the statistical power to detect differences between cases and controls due to the reduced sample size. Furthermore, 16S rRNA gene sequencing, as applied in our study, does not allow analyses on the strain level as it could lead to misclassifications [66]. Furthermore, the SPINGO classifier does not have a strategy to exclude sequencing errors, but the authors have shown that this shortcoming had little influence on SPINGO’s accuracy [67]. Another limitation resulting from the usage of 16S rRNA gene sequencing lays within the missing resolution on the strain level, forcing us to group metabolic capabilities of strains on the species level. As different strains of the same species may have different metabolic capabilities, computational modelling on the basis of the species level has to be treated with some care. Consequently, follow-up studies based on shotgun sequencing are needed to further corroborate our results and those found by other 16S RNA gene sequencing studies (Fig. 7). In this respect, it is noteworthy to mention that AGORA had a high coverage of the species mainly detected in the microbiome (Additional file 1: Table S1). However, AGORA does not show complete coverage, which presents a limitation to this study. Consequently, certain species and genera present in the microbiome may be excluded from analyses because they were not included in the AGORA collection. Further expansion of the microbial metabolic reconstruction collection is hence needed. Additionally, we could only extract species (and not strain) abundances from the sequenced samples, which have important consequences for metabolic modelling. The lack of strain-resolved taxonomic information required us to generate species-level metabolic models, in which we grouped the metabolic capabilities of multiple strains into one metabolic model of the correspondent species. This approach may overestimate the metabolic capabilities as not all biochemical reactions included in the species metabolic model may be present in a single strain.

Being cross-sectional in nature, causal inference is not possible. Consequently, although metabolic modelling has numerous times been shown to correctly predict attributes of living systems [68–70], our hypothesis on the role of *B. wadsworthia* in PD interlinking sulphur metabolism with disease severity requires experimental validation. Additionally, the computational prediction of secretion profiles is in need of validation via integration with stool and blood metabolome data. Furthermore, the statistical properties of populations of constraint-based metabolic models have not been described in detail so far. Basically, this study uses the predicted net production capacities as a further layer to the omics data, on which statistical screenings by sequential regressions can be performed in analogy to ‘ome-wide association studies. Furthermore, the host metabolism has not been considered in the current study but such analysis is computationally possible (e.g. [27, 71–74]. To this end, sex-specific whole-body metabolic models [26] have been developed, which include human physiological constraints, and which can be expanded with microbiome models to investigate potential host-microbiome metabolic interaction, but such analysis was beyond the scope of this study.

## Conclusion

Overall, this study represents a step towards a systems biology description of the metabolic consequences of PD-associated alterations in the microbiome, but further development of statistical and computational tools integrating omics data with modelling techniques, such as constraint-based modelling, will need to be done.

## Methods

### Description of the Luxembourg Parkinson’s Study

Data and biospecimen of the Luxembourg Parkinson’s Study cohort were utilised [30]. The Luxembourg Parkinson’s Study includes a variegated group of patients with typical PD, atypical parkinsonism, and secondary parkinsonism as well as healthy controls from Luxembourg and its neighbouring regions geographically defined as Great Region [30]. Within the cohort, healthy controls were selected amongst spouses of chosen patients and volunteers and individuals from other independent Luxembourgish studies [75, 76]. However, the corresponding information on the family relations between controls and cases was not available. Cancer diagnosis with ongoing treatment, pregnancy, and secondary parkinsonism in the frame of normotensive hydrocephalus were the exclusion criteria for enrolling in the patient or healthy control group. For 454 individuals (controls: *n* = 248, PD: *n* = 206) from the Luxembourg Parkinson’s Study cohort, stool samples were available and used for 16S RNA gene sequencing data (see below). As we aimed to target specifically typical PD, we excluded all individuals with age below 50 (controls: *n* = 47, PD: *n* = 9) and all individuals with an unclear status of PD diagnosis or an atypical PD diagnosis (PD: *n* = 47). PD patients were defined as typical PD, according to the inclusion criteria by the United Kingdom Parkinson’s Disease Society Brain Bank Clinical Diagnostic Criteria [77]. Furthermore, we excluded control patients with a UPDRS III score above 10, except for one control where the high UPDRS III score was caused by an arm injury. Furthermore, we excluded control persons who took dopaminergic medications (*n* = 5; intake for a different than anti-PD indication) and individuals who reported to have taken antibiotics in the last 6 months (controls: *n* = 20, PD: *n* = 13). Note that excluded observations behave sub-additive because of an overlap between the exclusion criteria (i.e. individuals below age 50 and taking antibiotics). Finally, from 309 individuals (controls: *n* = 162, cases: *n* = 147) fulfilling the inclusion criteria, 308 were included in statistical analyses as one individual had a missing BMI value.

All study participants gave written informed consents, and the study was performed in accordance with the Declaration of Helsinki. The Luxembourg Parkinson’s Study [30] was approved by the National Ethics Board (CNER Ref: 201407/13) and Data Protection Committee (CNPD Ref: 446/2017).

### Measurements and neuropsychiatric testing

All patients and healthy controls were assessed by a neurologist, neuropsychologist, or trained study nurse during the comprehensive battery of clinical assessment. Olfaction testing was conducted using the Sniffin’ Sticks 16-item version (SS) yielding the “Sniff Score”. Antibiotic usage was defined as intake of antibiotics within the previous 6 months prior to stool collection. Constipation was evaluated by the study neurologists based on the personal clinical story of each individual. As definition of constipation, two criteria were considered: (i) difficult stool passage includes straining, a sense of difficulty passing stool, incomplete evacuation, hard/lumpy stool, prolonged time to stool, or need for manual manoeuvres to pass stool [78], and (ii) symptom-based including fewer than three stools per week, stool form that is mostly hard or lumpy, and difficult stool passage (need to strain or incomplete evacuation) for more than 6 months [79]. For assessing PD-related motor and non-motor symptoms, the UPDRS rating scales I–IV were used [80]. The severity of the disease was reflected by the Hoehn and Yahr staging [81]. Non-motor symptoms were measured using a corresponding questionnaire NMS-PD [82]. The use of medication was recorded in details, and for this study, three classes of PD-specific medication was used: (1) levodopa, (2) dopamine receptor agonist, and (3) MAO-B/COMT inhibitors.

### Collection and processing of stool samples

All samples were processed following standard operating procedures [83, 84]: stool samples were collected at home by patients using the OMNIgene.GUT® kit (DNA Genotek) and sent to the Integrated Biobank Luxembourg (IBBL) where one aliquot of 1 ml was used for DNA extraction. The mean delay from sample collection to sample reception at IBBL was on average of 3.8 days. The microbiome profiles were obtained from stabilised samples within the OMNIgene®-GUT kit, which has been shown to be comparable for downstream 16S rRNA gene sequencing, robustness, and sample stability to the snap-frozen samples [84, 85]. Leaving the OMNIgene®-GUT-stabilised samples for 2 weeks at room temperature does not produce any significant effects on microbiome profiles [86, 87]. For the DNA extraction, a modified Chemagic DNA blood protocol was used with the MSM I instrument (PerkinElmer), the Chemagic Blood kit special 4 ml (Ref. CMG-1074) with a lysis buffer for faecal samples, and MSM I software. Samples were lysed using the SEB lysis buffer (included in the kit) and vortexed to obtain a homogenous suspension that was incubated for 10 min at 70 °C, then 5 min at 95 °C. Lysates (1.5 ml) were centrifuged for 5 min at 10,000*g* at RT. Supernatants were transferred to a 24XL deep-well plate. Plates were processed using the MSM I automated protocol.

### Analysis of the microbial composition with 16S rRNA gene sequencing

The V3–V4 regions of the 16S rRNA genes were targeted with gene-specific primers and sequenced at IBBL using an Illumina Platform (Illumina MiSeq) using 2 × 300 bp paired-end reads [30]. The primers were designed with Illumina overhang adapters and used to amplify templates from genomic DNA. Amplicons were generated, cleaned, indexed, and sequenced according to the Illumina-demonstrated 16S metagenomic sequencing library preparation protocol with certain modifications. In brief, an initial PCR reaction contained at least 12.5 ng of DNA. A subsequent limited-cycle amplification step was performed to add multiplexing indices and Illumina sequencing adapters. Libraries were normalised, pooled, and sequenced on the Illumina MiSeq system using 2 × 300 bp paired-end reads. Dual index barcoding was used using the Illumina Nextera XT index primers.

The demultiplexed samples were processed merging forward and reverse reads and quality filtered using the dedicated pipeline “Merging and Filtering tool (MeFit)” [88] with default parameters. To obtain a reliable microbial identification, identification to both genus and species taxonomic levels was obtained using the SPINGO (SPecies level IdentificatioN of metaGenOmic amplicons) classifier [67] with default parameters. The SPINGO classifier has been designed for species taxonomic assignment [67]. Relative abundances were computed, for each sample, parsing the classification results of the SPINGO classifier using an R (R Foundation for Statistical Computing, Vienna, Austria) [89] custom script. Briefly, for each sample, the counts of each genus/species were retrieved, and then the sum of the counts of all the genera/species was used to normalise to a total value of 1 each genus/species count. Information about the read counts can be found in Additional file 1: Table S3.

### Genome-scale metabolic reconstructions, flux balance analysis (FBA), and community metabolic modelling

A metabolic reconstruction consists of the list of all metabolic reactions known to occur in an organism and describe each reaction’s stoichiometry and directionality [22, 65]. Such reconstructions are generally generated from the genome of an organism, the corresponding genomic annotation, and extensive review of organism-specific, biochemical, and physiological literature [65]. These metabolic reconstructions can be visualised as networks (each reaction is an arch connecting the involved metabolites), and they can be converted in a computational format. When converting into a computational format, a sparse matrix named the stoichiometric matrix (*S*) is generated from the stoichiometric coefficients of each reaction. In the S matrix, each row represents a different metabolite in the network, and each column a different reaction [22]. By definition, each substrate of a reaction obtains a negative sign, while the product receives a positive sign. If a metabolite does not participate in a reaction, the stoichiometric coefficient, and thus the *S* matrix entry, is 0. Each metabolite variation over time (i.e. *dx*/*dt*) will be therefore obtained by the multiplication of *S* for a vector V containing the velocities (fluxes), at which each reaction happens, and a system of linear equations can be written [22].

Flux balance analysis (FBA) [22] is a method used to study the properties of the metabolic reconstructions. FBA is based on three assumptions: (i) Steady-state: under this assumption, no metabolite can be accumulated, and the change of concentration of each metabolite overtime is zero, and therefore, *S* × *v* = *dx*/*dt* = 0). (ii) Capacity constraints: the existence of a minimal and maximal flux allowed through a reaction represented as upper and lower bounds. These constraints can be obtained from experimental data (e.g. *v*_max_ of enzymes, dietary uptake rate). (iii) Objective function: the flux through one reaction, most commonly the biomass (growth) reaction [90], is optimised. These assumptions allow for the retrieval of one possible, but not necessarily unique, flux distribution through the network (encoded in the vector of fluxes *v*) that is consistent with all applied constraints. The objective value for the objective function is, in contrast, unique [22].

Metabolic reconstructions can provide mechanistic insight into the metabolism of single organisms under specific conditions. However, microbial communities are complex systems where the final result is given by the interaction of different microbes [91]. For this reason, a multispecies approach for metabolic modelling of microbial communities named compartmentalisation was developed [24, 73]. In compartmentalisation approaches, different reconstructions are joined through a common compartment allowing them to metabolically interact (shared metabolites). The microbiome modelling toolbox [24, 92] allows for the creation of personalised microbiota models, where hundreds of microbial reconstructions are joined on the base of their presence/absence in the relative sample. For each microbiota model, relative abundances are integrated into a community objective function joining the biomass contribution of each organism to the community. This method is specifically developed for compositional data as the sum of all the coefficients has to equal the value of 1. Then, metabolites secreted by each community overall are computed, which can then be absorbed by the human host or otherwise may be excreted in the faeces.

### Mapping detected species on the gut microbial reconstruction collection

Currently, strain-specific metabolic reconstructions have been published for 819 gut microbes, named the AGORA collection [23, 25, 46] corresponding to 646 species. In the analysis dataset of the current study (*n* = 309), 515 species were detected at least in 5% of the stools samples and 243 overlapped with AGORA. A total of 125 species were detected in at least 50% of the samples with an overlap of 87 AGORA species. Thus, 70% of the identified species were covered by the AGORA selection (Additional file 1: Table S1). We conclude that our AGORA collection covers most of the frequently found species in our dataset.

### Generation of personalised models

As a next step, we generated a generic microbiome metabolic reconstruction consisting of 257 microbial metabolic reconstructions, which were had a relative abundance in our dataset above 1e−4 and which were present in the AGORA collection. This generic microbiome reconstruction was then personalised to each sample by eliminating all species in a sample below this threshold (being 1e−4) and by adjusting the community biomass reaction coefficients to the normalised relative abundance data, as obtained with SPINGO [67]. In the absence of personal nutrition information, an average European diet was used to constrain each microbiome model [24, 25] (Additional file 1: Table S4). In average, the personalised microbiome models contained 67 species, 77,390 (non-unique) reactions, and 69,265 (non-unique) metabolites (Additional file 1: Table S2). Furthermore, on average, the personalised microbiome models covered 2727 unique reactions (Additional file 1: Table S2). The number of unique reactions, total reactions, and total metabolites was slightly higher in PD in comparison with controls.

### Analyses of diversity indices

All numerical ecology analyses were computed in R for both genus and species taxonomic resolution. Richness was computed as the total number of detected organisms, while the alpha diversity was computed using the Shannon index as implemented in the “diversity” function of the 2.5-2 R vegan package [93]. Pielou evenness was computed with the “diversity” function of the 2.5-2 R vegan package [93] using the Simpson index. The beta diversity was computed using the “vegdist” function of the 2.5-2 R vegan package [93] using the Bray-Curtis dissimilarity index. Possible differences between PD patients and controls for richness, Shannon diversity, and Pielou index were assessed using linear mixed models with the batch variable as the random effect variable and included age, BMI, sex, and read count variables as covariates, while the group variable was the predictor of interest. In exploratory post hoc analyses, we tested further whether any of the basic confounders (age, sex, and BMI) interacted with the group variable influencing the various diversity indices. For the beta diversity, we conducted ANOSIM and ADONIS analyses as implemented in the 2.5-2 R vegan package [93] using the functions “Adonis” and “Anosim” with default parameters.

### Analyses of relative abundances via fractional regressions

For descriptive statistics, metric variables were described by means and standard deviations, while nominal variables were described by proportions. Missing values were not imputed, and the pattern of missing values was not assessable via the ADA platform [30]. The read counts for each metagenomic feature (e.g. genera and species) were divided by total read counts such that relative abundances were retrieved. Relative abundances were checked for outliers. Observations with more than four standard deviations from the mean were excluded from the analyses. Only the genera and species detected in more than 50% of all samples were included in the analyses, resulting in 62 genera and 127 species.

The metagenomic data was analysed using fractional regressions as developed by [94]. Fractional regressions, developed in the field of econometrics, are part of the family of generalised linear models and are specifically designed for the analyses of fractional data, such as relative abundance data. The relative abundance is herein the response variable, which is then regressed on a vector of predictors. Fractional regressions are semiparametric methods designed to model fractional data without the need for specifying the distribution of the response variable. Moreover, fractional regressions are inherently robust against heteroscedasticity and against overdispersion. These characteristics make the method very suitable for the analysis of microbiome data, where different species may not be sampled from the same class of distributions. In general, the quasi log-likelihood ln *L* of fractional regressions is given by the Bernoulli log-likelihood function:

1 $$ \ln L=\sum \limits_{j=1}^N\left[{y}_j\ln \left(G\left({\boldsymbol{x}}_{\boldsymbol{j}}^{\prime}\beta \right)\right)+\left(1-{y}_j\right)\ln \left(1-G\left({\boldsymbol{x}}_{\boldsymbol{j}}^{\prime}\beta \right)\right)\right] $$

with *G*(***∗***) being a function fulfilling 0 ≤ *G*(***z***) ≤ 1 for all *z* ∈ *ℝ*, where ***x***_***j***_ represents the predictors for the individual *j*, *y*_*j*_ the fractional response variable in individual *j*, and *N* the sample size (see [94] for further details). The most canonical choice for *G*(***∗***) is the logistic function, which allows the interpretation of the regression coefficients as odds ratios (OR). In the case of microbiome data, an OR refers to the chance that a certain sequence read is assigned to certain species. For example, consider a case-control design, where in the cases, the mean relative abundance for a certain species was ${\overline{y}}_{\mathrm{cases}}=0.04$ and in the controls, ${\overline{y}}_{\mathrm{controls}}=0.02$. Then, a fractional regression using logit parametrisation with the relative abundance of the species as the response variable and the group variable as the predictor would result in:

2 $$ \mathrm{OR}=\frac{{\overline{y}}_{\mathrm{cases}}\left(1-{\overline{y}}_{\mathrm{controls}}\right)}{{\overline{y}}_{\mathrm{controls}}\left(1-{\overline{y}}_{\mathrm{cases}}\right)}=\frac{0.04\times 0.98}{0.96\times 0.02}=2.04. $$

Thus, in this example, we would state that the odds that a certain sequence read is assigned to this species is 2.04 times higher in cases than in controls. Now, as in other regression analyses, we can include covariates deriving estimates conditional on a set of variables for which we would like to control. Note that we understand the relative abundance herein as an estimate of the probability that a read is assigned to a certain species.

All fractional regressions included technical covariates, by which we mean batch (sequencing run), total read counts, and unclassified sequence read counts (reads for which a taxonomic assignment was not possible independently from any threshold of confidence estimate value used). The read count variables were included into the statistical model, as it has been shown that normalisation by division can introduce bias if certain statistical assumptions implied by the application of division are not fulfilled [95]. In the case of metagenomic data, the effect of read counts would be removed by division if the observations would be sampled from a multinomial distribution. However, this is not a given as species and genera correlate amongst each other, violating the assumptions needed to construct multinomial distributions. In consequence, read count normalisation by division is prone to introduce a bias into the metagenomic data; as potential bias, we corrected for by including the read counts as covariates into the model.

Before fitting the final statistical models, we explored the associations of basic covariates (i.e. age, sex, and BMI) with metagenomic features using fractional regressions as described above to avoid misspecifications of the statistical models. Since the data showed a broad range in age and BMI, we tested for potential non-linear associations by including these variables into the models as restricted cubic splines [96] using three knots defined by the 5% percentile, the median, and the 95% percentile. As in the case of age, we found species with indications of non-linear age associations with *p* < 0.01; age was modelled in all analyses via restricted cubic splines.

All *p* values are reported two-tailed. Statistical analyses were performed in STATA 14/MP (College Station, TX, USA). Summary statistics of the performed analyses are given in Additional files 2, 3, 4, and 5.

### Differences between PD and controls in microbial composition and the influence of covariates

To analyse the difference between genus abundances between PD and controls, fractional regressions were carried out with the relative abundance of the genus as the response variable, while including technical covariates, age (restricted cubic splines), sex, and BMI into the statistical modelling. The predictor of interest was the study group indicator variable. We corrected for multiple testing using the Benjamini-Hochberg procedure [97] by setting the false discovery rate (FDR) to 0.05. Consequently, we corrected for 62 tests when reporting genus results. These analyses were repeated analogously for the taxonomic level of species, while correcting for multiple testing via the FDR.

Next, we explored the possibility of statistical interactions between basic covariates (age, sex, and BMI) and the group indicator. For these analyses, we once again modelled age and BMI via restricted cubic splines allowing for non-linear interaction terms. We only tested two-way interaction terms. All interaction terms were introduced simultaneously into the statistical model and tested on significance via a Wald test [96], correcting for multiple testing via the FDR. For the globally significant test, the single interaction terms were investigated to explore which covariate-group interaction contributed to the overall significance. For interpretation, the interaction terms were visually inspected by plotting the predictions conditional on technical covariates. These analyses were then rerun with species abundances as a response variable instead of genus abundances.

We assessed the influence of constipation on the microbial composition. We introduced the binary predictor constipation (yes/no) as an additional predictor into the model and the corresponding group-constipation interaction term. Both terms were tested simultaneously on zero with a Wald test. The analyses were once again adjusted for technical covariates, age (restricted cubic splines), sex, and BMI, and we corrected for multiple testing via the FDR.

### Analyses of within PD phenotypes in relation to microbial composition

We investigated the association pattern of medication and clinical features regarding the microbial composition. These analyses were only performed on the IPD cases, while controls were excluded from the analyses. First, we analysed the disease duration as measured in years between the date of the stool sampling and the year of the diagnosis. The analyses were conducted as before via fractional regressions with the genus abundances as the response variable, while adjusting for technical covariates, age (restricted cubic splines), sex, and BMI. Then, we assessed in separate analyses the UPDRS III score as an indicator for motor symptoms, the non-motor symptoms as measured by the NMS, the Hoehn-Yahr staging of the disease as a global measure of disease progression, and the sniff score. All these analyses were performed adjusted for technical covariates, age (restricted cubic splines), sex, BMI, and disease duration. Each of these series of regression represents 62 tests, which were accounted for using the FDR. The impact of medication was analysed by examining three classes of medication: (a) levodopa, (b) mono-amino oxidase/catechol-*O*-methyltransferase inhibitors, and (c) dopamine receptor agonists. We generated three corresponding binary phenotypes (intake/no intake) and added these three variables simultaneously to the statistical model determining the significance of this add-on via a Wald test. We then tested each medication class in separate analyses, strictly correcting for multiple testing via the FDR (186 tests in total). The analyses were performed adjusted for technical covariates, age (restricted cubic splines), sex, BMI, and disease duration.

### Personalised constraint-based modelling of microbial communities

AGORA consists of a set of 818 strain-specific genome-scale metabolic reconstructions for microbes commonly found in the human gut [23, 46]. To match species taxonomic resolution, we combined the metabolites and biochemical reactions present in the strain-specific, metabolic reconstructions of the same species in one pan-species reconstruction (“panSpeciesModel.m”) using the function “createPanModels.m” of the microbiome modelling toolbox [24]. A pan-biomass reaction was built by averaging all strain-specific biomass reactions. A total of 646 species-specific metabolic reconstructions were assembled in this manner. Subsequently, we performed an automatic name matching between SPINGO species taxonomic assignment and panSpecies names. Note that we had to disregard all species that were not present within these 646 metabolic reconstructions but identified in the microbiome data (Additional file 1: Table S1). A threshold for assessing the bacterial presence of a relative abundance value of 0.0001 was used to reduce the time of computations while limiting the order of magnitude simulation results of stoichiometric coefficients to 10. A total of 259 species overlapped between our set of species models and SPINGO species assignment when considering species identified at least in 10% of the microbiome samples (Additional file 1, Table S1). In the next step, the retrieved microbial abundance information for each sample was integrated into a community modelling setup obtaining personalised microbiome models using the automated module of the microbiome modelling toolbox [24] called mgPipe within the COBRA toolbox [92] (commit: b097185b641fc783fa6fea4900bdd303643a6a7e). Briefly, the metabolic models of the community members are connected by a common compartment, where each model can secrete/uptake metabolites. An average European diet was applied as input constraints for the metabolite (diet) update reactions in each microbiome model [46]. The average European diet (cf. Additional file 1: Table S4) was extrapolated from an Austrian survey, which included 1002 participants from different ages [98]. A community objective function was formulated based on the sum of each microbial model objective function and constrained to a lower bound of 0.4 per day and upper bound of 1 per day, corresponding to a faecal excretion of once every 2.5 days to once a day. A set of exchange reactions connects the shared compartment to the environment enabling to predict metabolite uptake (from the defined diet) and secretion flux rates (metabolic profiles/NMPCs) consistent with the applied constraints. The personalisation of each microbiome model was achieved by adjusting stoichiometric coefficients in the community biomass reactions to each sample’s relative microbial abundance after removing undetected species from the community models.

Relative reaction abundances were calculated by summing the number of species having the reaction in a microbiome model and scaling the sum by the respective species relative abundance. Community metabolic profiles of these microbial communities were assessed using flux variability analysis on the exchange reactions [99]. AGORA microbial metabolic reconstructions used for the construction of the community models were downloaded from the VMH (www.vmh.life, [46]). All computations were performed in MATLAB version 2018a (Mathworks, Inc.), using the IBM CPLEX (IBM, Inc.) solver through the Tomlab (Tomlab, Inc.) interface.

### Statistical analyses of fluxes

The NMPCs were log-transformed such that the skewness of the distribution was minimised [100]. This type of transformation was applied because of the very differently skewed distributions of the single NMPCs. Then, outliers were excluded using the 4-SD outlier rule as before. Only fluxes with more than 50% non-zero values were retained in analyses. Furthermore, NMPCs with distributions not suitable for statistical analyses (e.g. distributions with a high number of observations with exact the same numerical value) were excluded resulting in 129 NMPCs included into the analyses.

The NMPCs were analysed with mixed linear regressions including the batch as random effects. Including the batch variable as a random effect has a higher statistical power in comparison with the fixed effect approach but relies on more restrictive assumptions. We tested the corresponding random effect assumption by Hausman specification tests and found no indications of violations of the random effects assumption. Note that this possibility to account for batch effects via random effects is not available with fractional regressions where batch effects were corrected via fixed effects.

We performed the same analyses as with the metagenomic data, with the sole exception of replacing the fractional regression model with the linear mixed model. In all other aspects, the analyses followed the same scheme. Multiple testing correction was performed using the FDR, correcting for 129 tests.

### Analyses of species contribution to fluxes

To investigate the contribution of species and genera, we calculated for all included genera and all analysed fluxes, the pairwise correlation, and the corresponding variance contribution (the squared correlation). We classified every correlation above 0.5 (equal to 25% of variance contribution) as a strong correlation in accordance with classical classifications of effect size [101].

## Supplementary information

**Additional file 1.** Extended results on microbial abundance analyses (Fig. S1, S2, and S3) and background information about characteristics of the community models and read counts (Tables S1, S2, S3, and S4).

**Additional file 2.** Summary statistics of the species phenotype association analyses.

**Additional file 3.** Summary statistics of the genus phenotype association analyses.

**Additional file 4.** Summary statistics for the variance contributions of genera to net production capacities.

**Additional file 5.** Summary statistics of the net production capacity phenotype association analyses.

## Data Availability

The datasets for this manuscript (16S rRNA sequences) are not publicly available as they are linked to the Luxembourg Parkinson’s Study and its internal regulations. Requests to access the datasets should be directed to Prof. Rejko Krueger, mean of contact via email: rejko.krueger@uni.lu. The mgPipe pipeline is available within the COBRA toolbox (https://github.com/opencobra/cobratoolbox), and the custom scripts with related documentation are available at the GitHub repository: https://github.com/ThieleLab/CodeBase/tree/master/ND_collect.
