# Supplementary material for: Parkinson’s disease-associated alterations of the gut microbiome predict disease-relevant changes in metabolic functions
Source: BMC Biol. 2020 Jun 9;18:62. doi: 10.1186/s12915-020-00775-7 (PMC7285525; doi:10.1186/s12915-020-00775-7)
Supplement: Supplementary file 1 — Additional file 1. Extended results on microbial abundance analyses (Fig. S1, S2, and S3) and background information about characteristics of the community models and read counts (Tables S1, S2, S3, and S4). [file 12915_2020_775_MOESM1_ESM.docx]

**Parkinson’s disease-associated alterations of the gut microbiome predict disease-relevant changes in metabolic functions**

Federico Baldini^1^*, Johannes Hertel^2,3^*, Estelle Sandt^4^, Cyrille C. Thinnes^2^, Lorieza Neuberger-Castillo^4^, Lukas Pavelka^1,5^, Fay Betsou^4^, Rejko Krüger^1,5,6^, Ines Thiele^1,2,7,8 #^ on behalf of the NCER-PD Consortium

* These authors contributed equally.

^1^ Luxembourg Centre for Systems Biomedicine (LCSB), University of Luxembourg, Campus Belval, Esch-sur-Alzette, Luxembourg.

^2^ School of Medicine, National University of Ireland, Galway, Ireland.

^3^ Department of Psychiatry and Psychotherapy, University Medicine Greifswald, Greifswald, Germany.

^4^ Integrated BioBank of Luxembourg, 1, Rue Louis Rech, 3555 Dudelange, Luxembourg

^5^ Parkinson Research Clinic, Centre Hospitalier de Luxembourg (CHL), Luxembourg

^6^ Transversal Translational Medicine, Luxembourg Institute of Health (LIH), Strassen, Luxembourg

^7^ Discipline of Microbiology, School of Natural Sciences, National University of Ireland, Galway, Ireland.

^8^ APC Microbiome, Ireland.

^#^ email: [ines.thiele@nuigalway.ie](mailto:ines.thiele@nuigalway.ie)

**Content**

**1 Supplementary Figures p.2-4**

**2 Supplementary Tables p.5-9**

**1 Supplementary Figures**


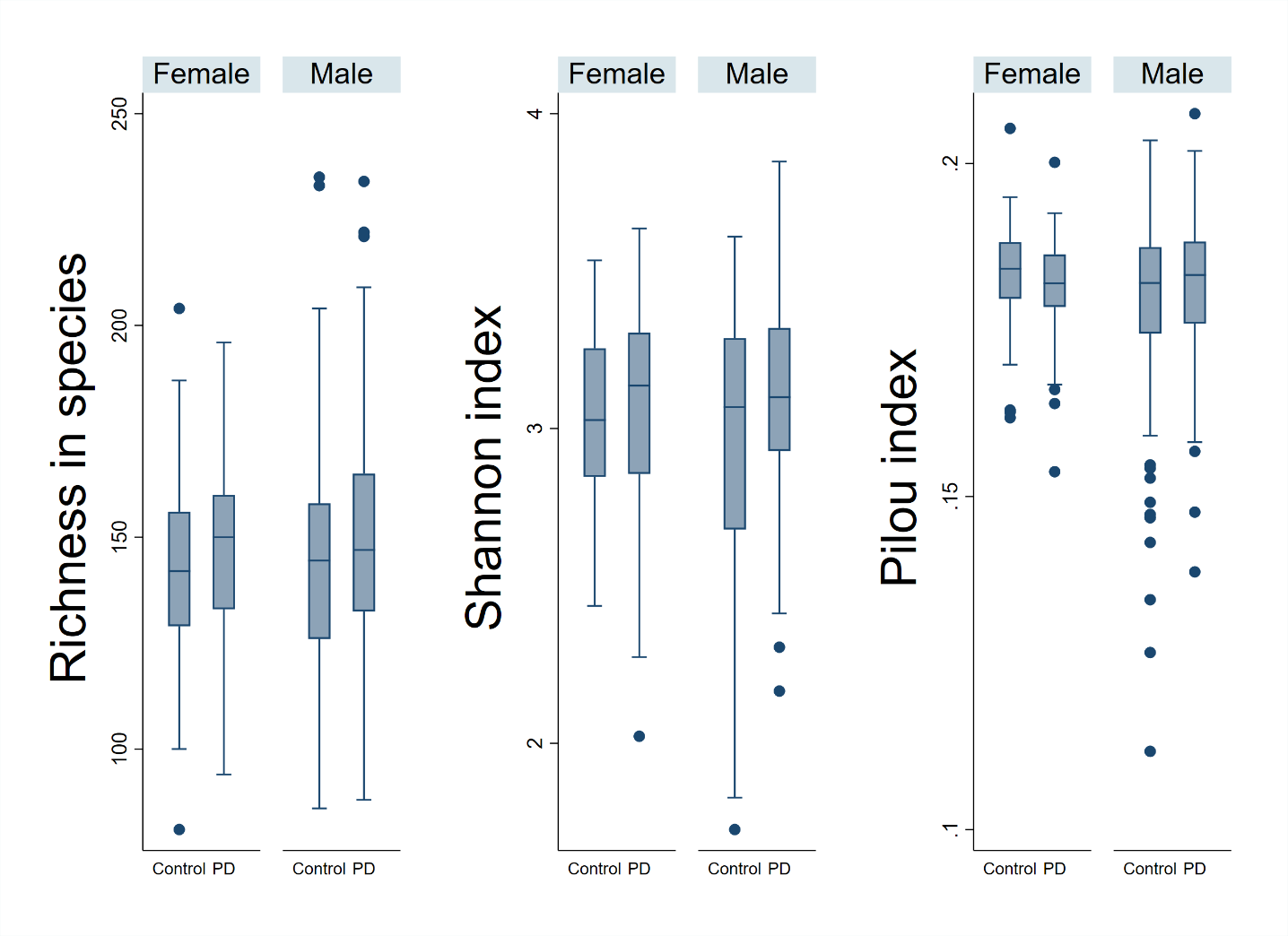


**Figure S1**: **Box plots for diversity indices in dependence on study group and sex.** Species richness was slightly increased in PD (regression coefficient b=4.76, 95%-Confidence interval (CI):0.44;9.08), p=0.03), while for the Shannon index no significant difference among the groups could be detected. For the Pilou index, we detected a significant sex-group interaction (p=0.043).


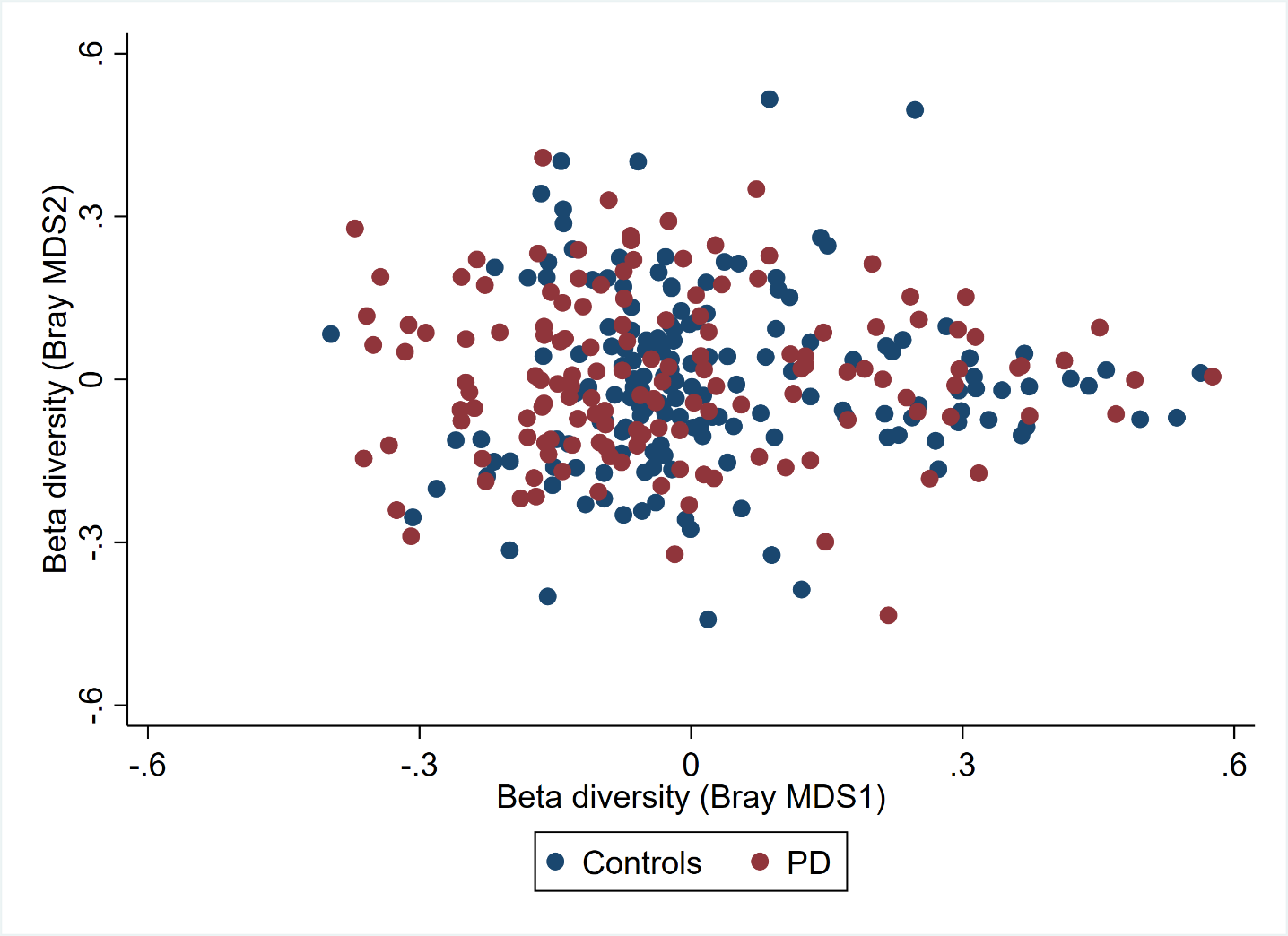


**Figure S2: Multidimensional scaling plot for the first and second dimensions for controls and PD patients.** While no major clustering was perceivable, ANOSIM detected significant but small differences (ANOSIM statistics R=0.04, p=0.001) between the two groups.


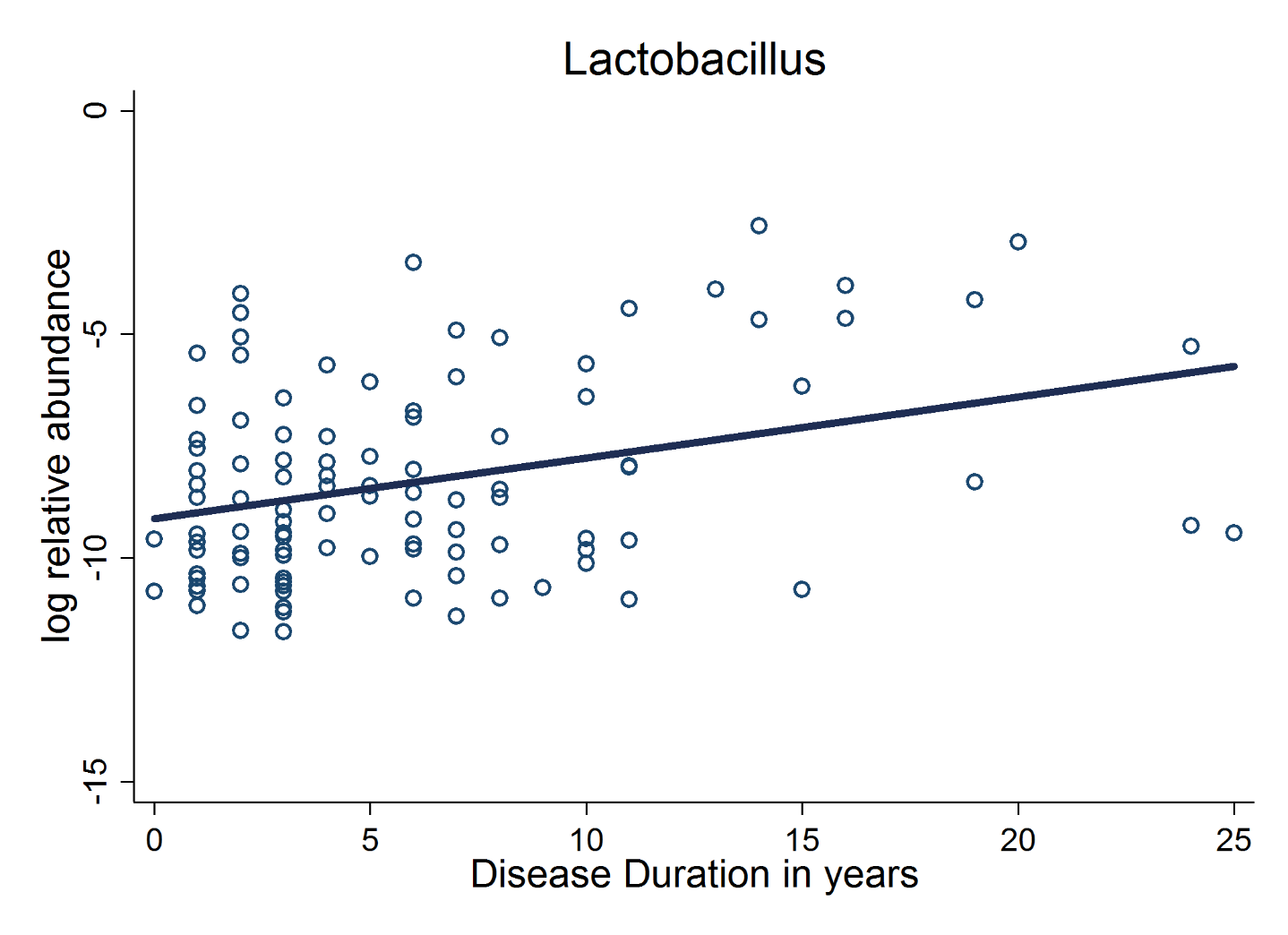


**Figure S3:** **Scatter plot of Lactobacillus relative abundance in dependence on disease duration** with corresponding linear regression line. The slope of the regression line is significantly bigger than zero (FDR<0.05).

**2 Supplementary Tables**

**Table S1:** **Number of species present in AGORA in dependency on the number of detected species in at least 5,50%,75%, and 100% of the samples.**

| **Including only species present in a subset, or all, microbiome samples (%)** | **Number of detected species** | **Number of detected species present in AGORA** |
| --- | --- | --- |
| 5 | 515 | 243 |
| 50 | 125 | 87 |
| 75 | 83 | 63 |
| 90 | 57 | 46 |
| 100 | 0 | 0 |

**Table S2**:  **Personalized microbiome metabolic model characteristics.**

| **Number of** | **Overall** | **PD** | **Control** |
| --- | --- | --- | --- |
| **Species** | 67 ± 11 | 69 ± 11 | 66 ± 10 |
| **Unique Reactions** | 2727 ± 96 | 2747 ± 101 | 2708 ± 86 |
| **Total Reactions** | 77390 ± 11853 | 79059 ± 12639 | 75875 ± 10933 |
| **Total Metabolites** | 69265 ± 10679 | 70757 ± 11353 | 67910 ± 9868 |

Mean ± Standard Deviation

**Table S3**:  **Descriptive statistics for read counts.**

|  | Overall | PD | Control |
| --- | --- | --- | --- |
| Total read counts | 124120.6 ±116053.4 | 125889.1±152762.6 | 122527.8 ± 116053.4 |
| Unclassified read counts species | 50816.67±54958.9 | 50997.6± 54591.1 | 50653.68 ± 55457.8 |
| Unclassified read counts genus | 35544.8 ± 37647.1 | 35380.5 ± 35812.8 | 35692.7 ± 39336.9 |

Mean ± Standard Deviation

**Table S4:** Uptake constraints implemented for simulation on an average European diet as derived from www.vmh.life.

| **Uptake reaction for** | **VMH ID** | **Lower bound** |
| --- | --- | --- |
| Ethanol | EX_etoh[e] | 234,4340155 |
| Water | EX_h2o[e] | 165892,3425 |
| Beta-Carotene | EX_caro[e] | 0,003585581 |
| Retinol | EX_retinol[e] | 3,002252387 |
| Thiamin | EX_thm[e] | 5,407858013 |
| Adenosylcobalamin | EX_adpcbl[e] | 3,60855E-06 |
| Riboflavin | EX_ribflv[e] | 0,003693234 |
| Nicotinate | EX_nac[e] | 0,308616192 |
| Nicotinamide | EX_ncam[e] | 0,1455276 |
| (R)-Pantothenate | EX_pnto_R[e] | 0,025569705 |
| Pyridoxamine | EX_pydam[e] | 0,00317177 |
| Pyridoxine | EX_pydxn[e] | 0,003172205 |
| Pyridoxal | EX_pydx[e] | 0,003210459 |
| Biotin | EX_btn[e] | 0,000178666 |
| 10-Formyltetrahydrofolate | EX_10fthf[e] | 0,000125752 |
| 5-Methyltetrahydrofolate | EX_5mthf[e] | 0,000129311 |
| 5, 6, 7, 8-Tetrahydrofolate | EX_thf[e] | 0,000133696 |
| L-Ascorbate | EX_ascb_L[e] | 0,583441166 |
| Calctriol | EX_vitd3[e] | 8,76149E-06 |
| Alpha-Tocopherol | EX_avite1[e] | 0,03685808 |
| Phylloquinone | EX_phyQ[e] | 0,000136877 |
| Calcium | EX_ca2[e] | 18,5490793 |
| Chloride | EX_cl[e] | 375,5574987 |
| Kalium | EX_k[e] | 83,43380658 |
| Mg | EX_mg2[e] | 14,52993211 |
| Sodium | EX_na1[e] | 172,4462717 |
| Phosphate | EX_pi[e] | 14,22660928 |
| Cu2+ | EX_cu2[e] | 0,035674944 |
| Iron (Fe2+) | EX_fe2[e] | 0,109248814 |
| Iron (Fe3+) | EX_fe3[e] | 0,109248814 |
| Mn2+ | EX_mn2[e] | 0,074702331 |
| Zinc | EX_zn2[e] | 0,150963597 |
| D-Mannitol | EX_mnl[e] | 0,170169038 |
| Xylitol | EX_xylt[e] | 0,141969085 |
| Lactose | EX_lcts[e] | 4,126714705 |
| Maltose | EX_malt[e] | 8,413758248 |
| Sucrose | EX_sucr[e] | 202,3830217 |
| D-Fructose | EX_fru[e] | 106,5058652 |
| D-Galactose | EX_gal[e] | 0,029974039 |
| Cellulose | EX_cellul[e] | 0,052346876 |
| L-Alanine | EX_ala_L[e] | 37,20856362 |
| L-Arginine | EX_arg_L[e] | 20,3518771 |
| L-Aspartate | EX_asp_L[e] | 47,08674913 |
| L-Cysteine | EX_cys_L[e] | 7,511831638 |
| L-Glutamate | EX_glu_L[e] | 103,1222005 |
| Glycine | EX_gly[e] | 36,76468629 |
| Urate | EX_urate[e] | 2,919392803 |
| L-Histidine | EX_his_L[e] | 14,02407663 |
| L-Isoleucine | EX_ile_L[e] | 28,92167513 |
| L-Leucine | EX_leu_L[e] | 46,88780991 |
| L-Lysine | EX_lys_L[e] | 33,31154825 |
| L-Methionine | EX_met_L[e] | 10,72740469 |
| L-Phenylalanine | EX_phe_L[e] | 20,71498664 |
| L-Proline | EX_pro_L[e] | 53,81527918 |
| L-Serine | EX_ser_L[e] | 37,37922556 |
| L-Threonine | EX_thr_L[e] | 25,88105024 |
| L-Tryptophan | EX_trp_L[e] | 4,554824772 |
| L-Tyrosine | EX_tyr_L[e] | 15,64685397 |
| L-Valine | EX_val_L[e] | 37,78437731 |
| Decanoate (N-C10:0) | EX_dca[e] | 10,74842619 |
| Laurate | EX_ddca[e] | 13,45904717 |
| Tetradecanoate (N-C14:0) | EX_ttdca[e] | 30,21138004 |
| Tetradecenoate (N-C14:1) | EX_ttdcea[e] | 3,783984795 |
| Pentadecanoate | EX_ptdca[e] | 2,89407663 |
| Hexadecanoate (N-C16:0) | EX_hdca[e] | 92,78506719 |
| Heptadecanoate | EX_hpdca[e] | 1,779524923 |
| Octadecanoate (N-C18:0) | EX_ocdca[e] | 35,85086639 |
| Octadecenoate (N-C18:1) | EX_ocdcea[e] | 101,5927326 |
| Linoleic Acid (All Cis C18:2) | EX_lnlc[e] | 54,98546187 |
| Alpha-Linolenic Acid | EX_lnlnca[e] | 4,163336898 |
| Stearidonic Acid | EX_strdnc[e] | 0 |
| Arachidate | EX_arach[e] | 1,033055397 |
| 11-Cis-Eicosenoate | EX_CE2510[e] | 1,086665034 |
| Cis, Cis-11, 14-Eicosadienoyl Coenzyme A | EX_CE4843[e] | 0,114865263 |
| Nc20:4 | EX_arachd[e] | 0,536810751 |
| Behenic Acid | EX_docosac[e] | 0,454096231 |
| Docosenoic Acid (C22:1) | EX_doco13ac[e] | 0,560730443 |
| Adrenic Acid | EX_adrn[e] | 0 |
| Clupanodonic Acid (Docosapentaenoic (N-3)) | EX_clpnd[e] | 0,048376886 |
| Nc22:6 | EX_crvnc[e] | 0,16630008 |
| Butyrate (N-C4:0) | EX_but[e] | 26,33425644 |
| Octanoate (N-C8:0) | EX_octa[e] | 6,845572908 |
| Cholesterol | EX_chsterol[e] | 1,23218851 |
| D-Sorbitol | EX_sbt_D[e] | 0,032935943 |
| D-Glucose | EX_glc_D[e] | 135,5803501 |
| Lignoceric Acid | EX_lgnc[e] | 0,222615856 |
| Folate | EX_fol[e] | 0,000134614 |
| Hexadecenoate (N-C16:1) | EX_hdcea[e] | 7,120473022 |
| Starch, Structure 1 (1, 6-{7[1, 4-Glc], 4[1, 4-Glc]}) | EX_strch1[e] | 33,03699973 |
| Iodide | EX_i[e] | 0,00201238 |
| Starch exchange | EX_starch1200[e] | 0,30586977 |
